# Systems genetics analysis of human body fat distribution genes identifies adipocyte processes

**DOI:** 10.26508/lsa.202402603

**Published:** 2024-05-03

**Authors:** Jordan N Reed, Jiansheng Huang, Yong Li, Lijiang Ma, Dhanush Banka, Martin Wabitsch, Tianfang Wang, Wen Ding, Johan LM Björkegren, Mete Civelek

**Affiliations:** 1 Department of Biomedical Engineering, University of Virginia , Charlottesville, VA, USA; 2 Center for Public Health Genomics, University of Virginia , Charlottesville, VA, USA; 3 Novo Nordisk Research Center China, Novo Nordisk A/S, Beijing, China; 4 Department of Genetics and Genomic Sciences, Icahn School of Medicine at Mount Sinai , New York, NY, USA; 5 Division of Paediatric Endocrinology and Diabetes, Department of Paediatrics and Adolescent Medicine, Ulm University Medical Centre, Ulm, Germany; 6 Department of Medicine, Karolinska Institutet, Huddinge, Stockholm, Sweden

## Abstract

Excess abdominal fat is a sexually dimorphic risk factor for cardio-metabolic disease and is approximated by the waist-to-hip ratio adjusted for body mass index (WHR_adjBMI_). Whereas this trait is highly heritable, few causal genes are known. We aimed to identify novel drivers of WHR_adjBMI_ using systems genetics. We used two independent cohorts of adipose tissue gene expression and constructed sex- and depot-specific Bayesian networks to model gene-gene interactions from 8,492 genes. Using key driver analysis, we identified genes that, in silico and putatively in vitro, regulate many others. 51–119 key drivers in each network were replicated in both cohorts. In other cell types, 23 of these genes are found in crucial adipocyte pathways: Wnt signaling or mitochondrial function. We overexpressed or down-regulated seven key driver genes in human subcutaneous pre-adipocytes. Key driver genes *ANAPC2* and *RSPO1* inhibited adipogenesis, whereas *PSME3* increased adipogenesis. *RSPO1* increased Wnt signaling activity. In differentiated adipocytes, MIGA1 and UBR1 down-regulation led to mitochondrial dysfunction. These five genes regulate adipocyte function, and we hypothesize that they regulate fat distribution.

## Abstract (precis)

Using Bayesian network analysis, we modeled gene-gene interactions and predicted novel regulators of adipose tissue fat storage. We show that five novel genes affect adipogenesis or mitochondrial function in human fat cells.

## Introduction

Obesity (measured as body mass index [BMI]) is a condition that affects roughly 40% of Americans (Hales et al, 2020) and significantly increases the risk of cardio-metabolic disease (Riaz et al, 2018; Cheng et al, 2019). Excess energy is primarily stored as lipids in two large adipose tissue depots, abdominal visceral and lower body subcutaneous. Adipocytes in each depot can expand by increasing their number (through pre-adipocyte proliferation and differentiation) or their size (through adipocyte glucose and free fatty acid uptake, lipogenesis, and lipolysis) to increase storage capacity (Drolet et al, 2008; Haczeyni et al, 2018). Whereas subcutaneous adipocytes expand to store lipids safely and efficiently (Williams et al, 1997), visceral adipocytes are less able to accommodate excess energy through adipocyte expansion (Tchkonia et al, 2005; Pellegrinelli et al, 2016). This causes inflammation (Alvehus et al, 2010; Samaras et al, 2010; Després, 2012), insulin resistance (Kabir et al, 2005), hypertension (Mathieu et al, 2009), and negative effects on lipid metabolism (Lemieux et al, 2001) in other tissues. Thus, disproportionate visceral fat storage contributes to the disease risk imparted by over-nutrition. Multiple studies find that the ratio of waist circumference to hip circumference (WHR), an approximation of human fat distribution, and WHR independent of overall obesity (WHR_adjBMI_), are both similar or better predictors for cardiovascular disease risk and Type II Diabetes risk than BMI (Schleinitz et al, 2014a; Dale et al, 2017; Emdin et al, 2017). Males generally have higher WHR_adjBMI_ and higher disease risk than females (Williams et al, 1997; Bots et al, 2017; Ye et al, 2019). Our understanding of the genetic and molecular mechanisms that contribute to fat distribution is limited, and thus, unlike overall obesity (Drucker, 2018; Samms et al, 2021), no targeted therapeutics or lifestyle interventions to combat abdominal obesity are known (Kesztyüs et al, 2018).

To date, only five genes (*KLF14*, *LRP5*, *TBX15*, *RSPO3*, *SHOX2*) are mechanistically linked to disproportionate fat storage in one depot compared with the other. They affect pathways crucial to the expansion of subcutaneous and visceral adipocyte populations (Gesta et al, 2006, 2011; Lee et al, 2013; Loh et al, 2015, 2020; Small et al, 2018; Yang et al, 2022). *LRP5* and *RSPO3* affect adipocyte differentiation by controlling Wnt signaling, whereas *TBX15* controls adipocyte differentiation and mitochondrial function.

The Wnt signaling pathway is a well-established driver of cell fate, differentiation, and proliferation in many cell types (Teo & Kahn, 2010); Wnt inhibits adipogenic differentiation by transcriptionally up-regulating osteogenic genes when down-regulating *PPARɣ* and *CEBPα* (de Winter & Nusse, 2021). In many contexts, the non-canonical Ca^2+^ form of Wnt signaling is inhibitory of the canonical Wnt pathway (Akoumianakis et al, 2022). Wnt signaling activity is positively associated with visceral adiposity (Fuster et al, 2015; Chen & Wang, 2018), and many Wnt pathway genes, especially ligands and receptors used in Ca^2+^ non-canonical Wnt signaling, are differentially expressed between fat depots (Zuriaga et al, 2017).

Mitochondrial function correlates strongly with cardio-metabolic diseases and can alter adipogenic differentiation (Lee et al, 2019). In mature adipocytes, mitochondria can facilitate physical connections with the lipid droplet (Cui & Liu, 2020), dissipate excess energy via thermogenesis through UCP1 (Montanari et al, 2017), and promote lipid homeostasis (Lee et al, 2019). Human visceral fat has higher mitochondrial activity compared with subcutaneous fat (Pafili et al, 2022), and in multiple metabolic disease states, only visceral mitochondria become dysregulated (Kraunsøe et al, 2010; Pafili et al, 2022). We hypothesize that other putative drivers of fat distribution affect Wnt signaling or mitochondrial function in adipocytes, with different outcomes in each depot.

Many genes potentially contribute to fat distribution. WHR_adjBMI_ is a complex trait that is up to 60% heritable (Schleinitz et al, 2014a), and recent genome-wide association study (GWAS) meta-analyses have uncovered ∼350 loci associated with WHR_adjBMI_. Approximately one-third of the GWAS loci are associated with fat distribution in only one sex (Pulit et al, 2017, 2019), and some have greater magnitude of association in one sex (Winkler et al, 2015). Many of the ∼500 genes near these loci have active regulatory regions in adipose tissue (Shungin et al, 2015) and are highly expressed in adipose tissue, many with differential expression between depots (Karastergiou et al, 2013; Schleinitz et al, 2014b). The expression levels of many of these genes are associated with genetic variants within the loci, which are termed expression quantitative trait loci (eQTLs) (Civelek et al, 2017; Raulerson et al, 2019; Wu et al, 2019). Single-gene defects that cause lipodystrophy, an extreme fat distribution phenotype, also affect adipocyte function (Lightbourne & Brown, 2017).

Computational models that describe gene-gene interactions, such as networks, can be powerful tools for interrogating regulatory relationships between many genes at once and prioritizing biologically relevant regulators in the geneset (Schadt & Lum, 2006; Civelek & Lusis, 2014). Correlation-based networks, which identify groups of co-regulated genes (Langfelder & Horvath, 2008), are frequently used to nominate disease regulators, as well as to annotate genes of unknown function or identify conserved gene programs (van Dam et al, 2018). Bayesian networks improve upon correlation-based networks by modeling causal, directed connections, and have been used to understand gene-gene interactions in many human disease models (Zhang et al, 2013, 2020; Hamed et al, 2015; Huan et al, 2015; McKenzie et al, 2017; Shu et al, 2017, 2019; Agrahari et al, 2018; Chella Krishnan et al, 2018; Carcamo-Orive et al, 2020; Hartman et al, 2021). The directed network structure allows researchers to easily nominate in silico “key drivers” of tissue-specific gene regulation and of disease (Sieberts & Schadt, 2007). Network key driver genes are putatively regulators of tissue- or disease-relevant processes. Whereas this process allows us to prioritize certain GWAS candidate genes over others, it can also nominate candidate regulators that are not genetically regulated. In various cell types and disease models, these key driver genes, both GWAS candidates and other mechanistic genes, are biologically relevant and alter disease-related pathways in vitro (Zhang et al, 2013, 2020; Mäkinen et al, 2014; Hamed et al, 2015; Huan et al, 2015; McKenzie et al, 2017; Shu et al, 2017, 2019; Agrahari et al, 2018; Chella Krishnan et al, 2018; Carcamo-Orive et al, 2020; Hartman et al, 2021).

**Figure 1.**
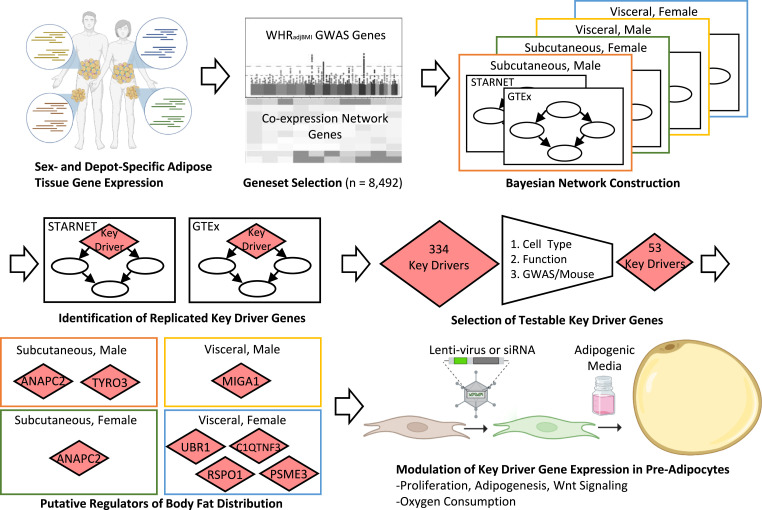
Overview of network construction, key driver gene identification, and functional validation. Publicly available RNA-seq gene expression data. The Genotype-Tissue Expression Project and Stockholm-Tartu Atherosclerosis Reverse Network Engineering Task (Franzén et al, 2016), from subcutaneous and visceral adipose tissue were subsetted between males and females. Co-expression network genes and WHR_adjBMI_ genome-wide association study (Pulit et al, 2019) genes used to construct Bayesian networks representing each sex and depot using RIMBANET (Zhu et al, 2008). Key driver genes shared between the Stockholm-Tartu Atherosclerosis Reverse Network Engineering Task and Genotype-Tissue Expression project were identified in each network. 53 key driver genes expressed in (pre-)adipocytes but unstudied in adipose tissue were prioritized for further study. Seven selected key driver genes identified were perturbed in human pre-adipocyte cells, and functional readouts of adipogenesis, Wnt signaling, proliferation, and mitochondrial oxygen consumption were collected.

To identify and prioritize candidate genes involved in fat distribution, we harnessed the predictive power of the Bayesian network construction and key driver analysis approach to identify genes likely to drive fat storage in subcutaneous and visceral fat (Fig 1). Because of the observed differences in body fat distribution between males and females, we constructed separate Bayesian networks of each sex-depot pair to model the distinct gene regulation in each tissue. To increase the predictive power of key driver analysis, we identified key driver genes replicated in two independent cohorts. We used publicly available data to prioritize key driver genes expressed in (pre-)adipocytes, unstudied in adipose tissue, and associated with fat storage in humans or mice. We focused on prioritized key drivers that affect Wnt signaling or mitochondrial function in other cell types, and we functionally profiled seven genes in subcutaneous pre-adipocytes.

## Results

### Bayesian networks model adipose tissue gene connections in a sex- and depot-specific manner

We interrogated two independent datasets of subcutaneous and visceral adipose tissue gene expression, Genotype-Tissue Expression project (GTEx) (The Genotype-Tissue Expression, 2020) and the Stockholm-Tartu Atherosclerosis Reverse Network Engineering Task (STARNET) (Franzén et al, 2016) (see the Materials and Methods section) and stratified each dataset by sex. Each dataset has about twice as many males as females (Table 1).

**Table 1.** Adipose tissue donor characteristics.

	Subcutaneous				Visceral			
	male		female		male		female	
	STARNET	GTEx	STARNET	GTEx	STARNET	GTEx	STARNET	GTEx
Samples	387	444	162	219	362	370	147	171
Age	64.3 ± 9.1	53.0 ± 12.8	69.2 ± 7.0	52.5 ± 12.7	64.4 ± 9.0	53.2 ± 12.8	69.2 ± 6.7	51.2 ± 12.6
BMI	28.7 ± 4.3	27.7 ± 4.1	29.5 ± 4.8	26.6 ± 4.2	28.7 ± 4.2	27.5 ± 4.0	29.5 ± 4.8	26.9 ± 4.2

We chose a set of 8,492 genes expressed in these datasets as network inputs, including 495 genes near WHR_adjBMI_ GWAS loci (see the Materials and Methods section, Table S1). We also added prior information about some genes to improve network performance, including genes with eQTLs in the corresponding adipose tissue depot (Table S2). We constructed eight sex- and depot-specific networks (see the Materials and Methods section) that contained an average of 6,250 genes connected, with an average of 6,821 directed edges between those genes (Tables 2 and S3). Each network displayed scale-free and small-world properties consistent with known biological networks (see the Materials and Methods section, Table S4).

Table S1 Shared network input genes.

Table S2 Tissue-Specific eGenes from Stockholm-Tartu Atherosclerosis Reverse Network Engineering Task and Genotype-Tissue Expression project, used as prior information during Bayesian Network construction.

**Table 2.** Sex- and depot-specific adipose tissue Bayesian network attributes and shared key driver genes.

	Subcutaneous				Visceral			
	male		female		male		female	
	STARNET	GTEx	STARNET	GTEx	STARNET	GTEx	STARNET	GTEx
Genes	7,360	6,066	6,814	5,560	7,343	6,402	5,908	4,546
Edges	9,174	6,782	6,865	5,651	8,752	6,845	5,962	4,533
Key drivers	833	695	753	632	779	670	660	502
Shared key drivers	119		88		119		51

Table S3 Edges in each constructed Bayesian network.

Table S4 Biological properties of constructed networks.

### Bayesian network structure identifies putative sex- and depot-specific “key drivers” of adipose tissue function and disease

Using our model of gene-gene interactions, we identified genes which are regulatory of many others using an approach called key driver analysis (Zhang et al, 2013; Shu et al, 2016). In each network, we identified key driver genes as those that regulated more downstream genes than expected by chance, or those that regulated more downstream genes that were also WHR_adjBMI_ candidate GWAS genes than expected by chance (see the Materials and Methods section, Fig 1). Others find that key drivers genes are more likely than other network features to be reproduced in independent datasets (Cohain et al, 2017) and have been experimentally validated for their regulatory role in cells (Zhang et al, 2013; Mäkinen et al, 2014). We identified an average of 691 key driver genes per network (Tables 2 and S5), and we hypothesize that some may regulate fat storage in adipocytes. To avoid problems of overfitting, we compared the key driver genes between the corresponding GTEx and STARNET networks. We identified 334 shared key driver genes in total (Tables 2, S5, and S6). Only 38 shared key driver genes were found in multiple sex-depot groups, and only one key driver, *ARHGEF12*, was identified in all eight networks.

Table S5 Key driver identification in all networks.

Table S6 Prioritized key driver genes. Networks MV, male visceral; FV, female visceral; MSQ, male subcutaneous; FSQ, female subcutaneous.

### Overlaps between STARNET and GTEx network key driver genes are significant

**Figure S1.**
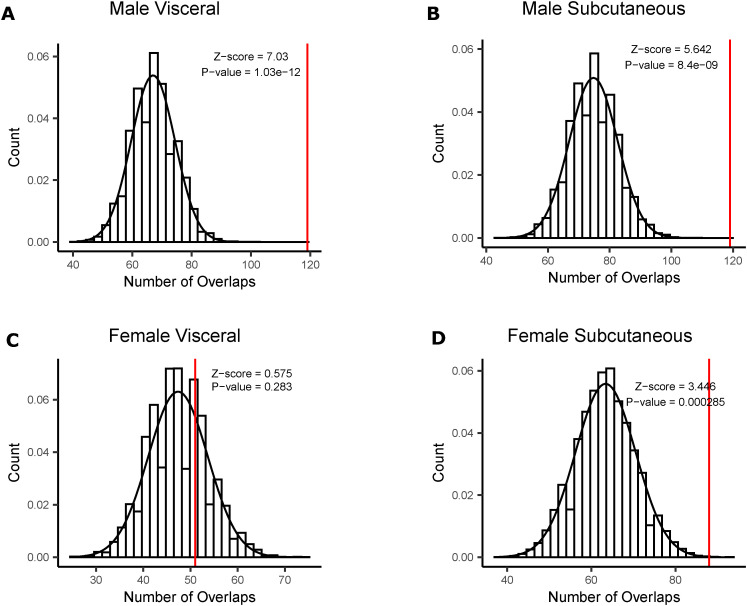
Significance of number of shared key drivers identified. **(A)** Key drivers shared between Stockholm-Tartu Atherosclerosis Reverse Network Engineering Task (STARNET) and Genotype-Tissue Expression project (GTEx) male subcutaneous networks (red), compared with a null distribution the number of shared genes between two randomly selected sets (black). **(B)** Key drivers shared between STARNET and GTEx male visceral networks (red), compared with a null distribution the number of shared genes between two randomly selected sets (black). **(C)** Key drivers shared between STARNET and GTEx female subcutaneous networks (red), compared with a null distribution the number of shared genes between two randomly selected sets (black). **(D)** Key drivers shared between STARNET and GTEx female visceral networks (red), compared with a null distribution the number of shared genes between two randomly selected sets (black). Data Information: Red line indicates the number of shared key drivers identified in Bayesian networks. Histogram represents a null distribution of the number of shared genes between two randomly selected sets. Randomly, sampled genes were chosen 10,000 times.

We observed that our networks constructed using male data were consistently larger than their female counterparts, with more connected nodes and edges. They had greater predictive power, identifying more genes that were key drivers, and further, the GTEx and STARNET male networks agreed more often, identifying more shared key drivers than female networks. To determine if this overlap between STARNET and GTEx was more than expected by chance, we chose a random set of genes from each network equal to the number of key drivers identified. We compared the shared genes in the random sets 10,000 times to form a null distribution. For the male subcutaneous and male visceral networks, we found that the number of shared key driver genes was significantly greater than random chance (Fig S1A and B). There were more female subcutaneous network key drivers than expected by chance, although the difference was less significant than in male networks, and there were not more female visceral network key drivers than random selection (Fig S1C and D).

**Figure S2.**
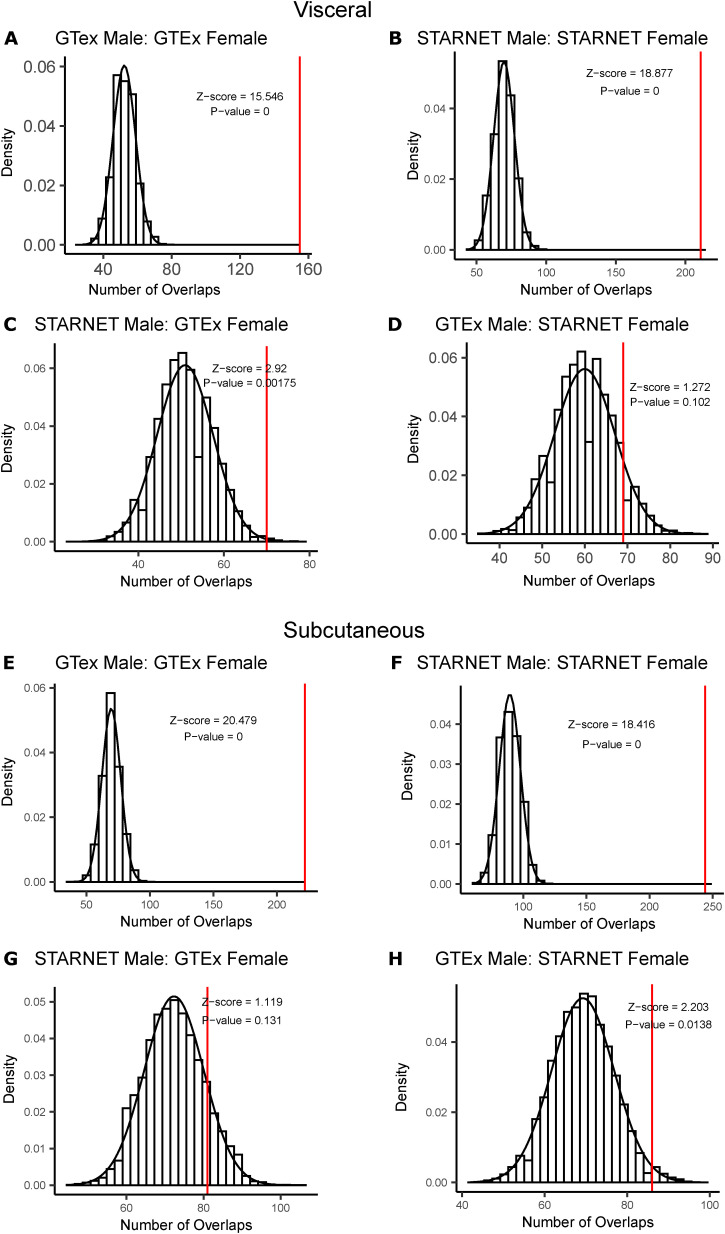
Significance of number of shared key drivers identified between non-similar groups. **(A)** Key drivers shared between visceral Genotype-Tissue Expression project (GTEx) male and visceral GTEx female networks (red), compared with a null distribution the number of shared genes between two randomly selected sets (black). **(B)** Key drivers shared between visceral Stockholm-Tartu Atherosclerosis Reverse Network Engineering Task (STARNET) male and visceral STARNET female networks (red), compared with a null distribution the number of shared genes between two randomly selected sets (black). **(C)** Key drivers shared between visceral STARNET male and visceral GTEx female networks (red), compared with a null distribution the number of shared genes between two randomly selected sets (black). **(D)** Key drivers shared between visceral GTEx male and visceral STARNET female networks (red), compared with a null distribution the number of shared genes between two randomly selected sets (black). **(E)** Key drivers shared between subcutaneous GTEx male and visceral GTEx female networks (red), compared with a null distribution the number of shared genes between two randomly selected sets (black). **(F)** Key drivers shared between subcutaneous STARNET male and visceral STARNET female networks (red), compared with a null distribution the number of shared genes between two randomly selected sets (black). **(G)** Key drivers shared between subcutaneous STARNET male and visceral GTEx female networks (red), compared with a null distribution the number of shared genes between two randomly selected sets (black). **(H)** Key drivers shared between subcutaneous GTEx male and visceral STARNET female networks (red), compared with a null distribution the number of shared genes between two randomly selected sets (black). Data Information: Red line indicates the number of shared key drivers identified in Bayesian networks. Histogram represents a null distribution of the number of shared genes between two randomly selected sets. Randomly, sampled genes were chosen 10,000 times.

To further interrogate the differences in network predictive power because of sex, depot, and dataset, we looked at the number of shared key driver genes between all other network pairs. The largest number of key drivers was shared between networks from the same dataset, which was significantly more than expected by chance (Figs S2A, B, E, and F and S3A, B, E, and F). When comparing networks from different datasets, the number of shared key drivers was only significantly more than random in some of the comparisons, and had much lower *P*-values (Figs S2C, D, G, and H and S3C, D, G, and H).

**Figure S3.**
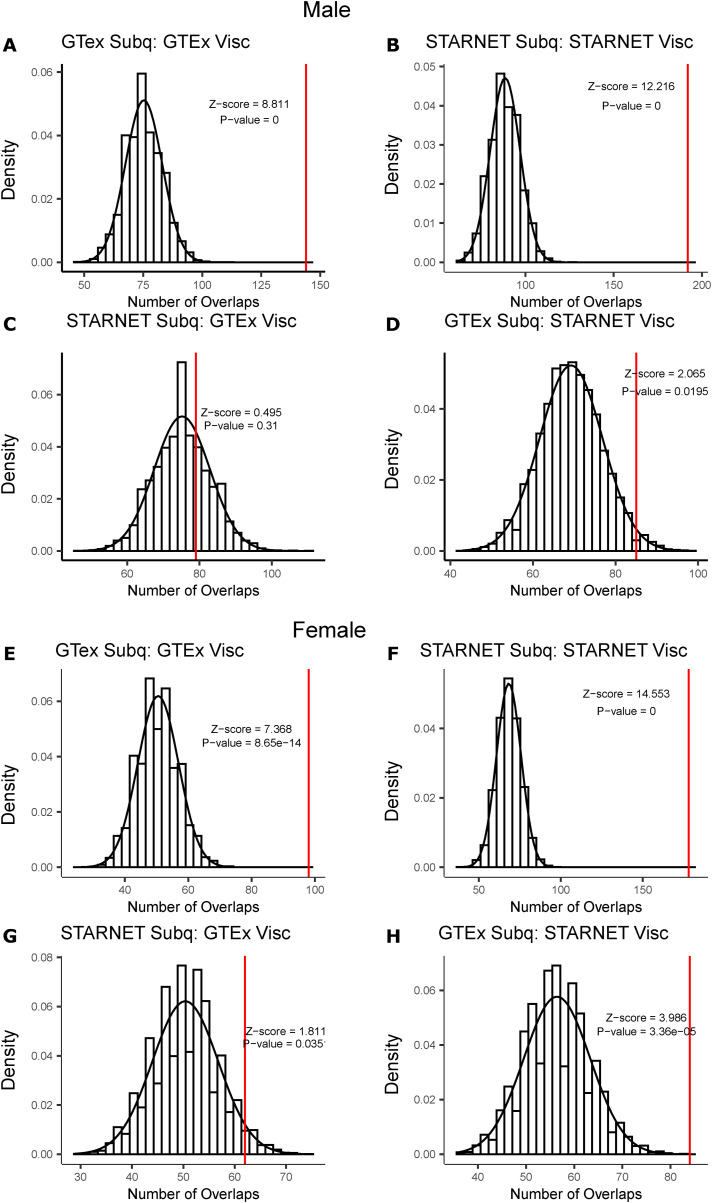
Significance of number of shared key drivers identified between non-similar groups. **(A)** Key drivers shared between male Genotype-Tissue Expression project (GTEx) subcutaneous and male GTEx visceral networks (red), compared with a null distribution the number of shared genes between two randomly selected sets (black). **(B)** Key drivers shared between male Stockholm-Tartu Atherosclerosis Reverse Network Engineering Task (STARNET) subcutaneous and male STARNET visceral networks (red), compared with a null distribution the number of shared genes between two randomly selected sets (black). **(C)** Key drivers shared between male STARNET subcutaneous and male GTEx visceral networks (red), compared with a null distribution the number of shared genes between two randomly selected sets (black). **(D)** Key drivers shared between male GTEx subcutaneous and male STARNET visceral networks (red), compared with a null distribution the number of shared genes between two randomly selected sets (black). **(E)** Key drivers shared between female GTEx subcutaneous and female GTEx visceral networks (red), compared with a null distribution the number of shared genes between two randomly selected sets (black). **(F)** Key drivers shared between female STARNET subcutaneous and female STARNET visceral networks (red), compared with a null distribution the number of shared genes between two randomly selected sets (black). **(G)** Key drivers shared between female STARNET subcutaneous and female GTEx visceral networks (red), compared with a null distribution the number of shared genes between two randomly selected sets (black). **(H)** Key drivers shared between female GTEx subcutaneous and female STARNET visceral networks (red), compared with a null distribution the number of shared genes between two randomly selected sets (black). Data Information: Red line indicates the number of shared key drivers identified in Bayesian networks. Histogram represents a null distribution of the number of shared genes between two randomly selected sets. Randomly, sampled genes were chosen 10,000 times.

**Figure S4.**
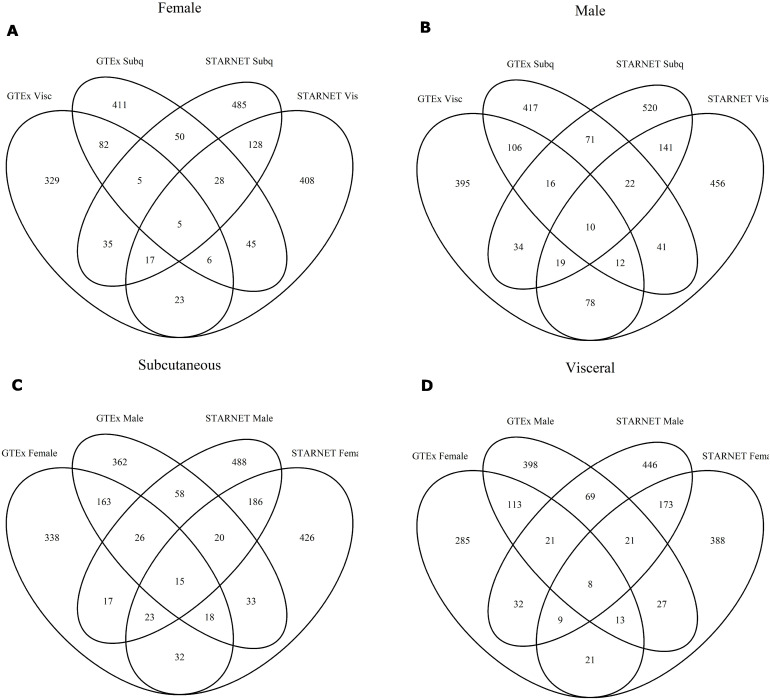
Key driver genes identified in each network. **(A)** Venn diagram comparing the key drivers from the four networks built on female expression data. **(B)** Venn diagram comparing the key drivers from the four networks built on male expression data. **(C)** Venn diagram comparing the key drivers from the four networks built on subcutaneous expression data. **(D)** Venn diagram comparing the key drivers from the four networks built on visceral expression data.

Finally, we compared the identity of the key drivers in each network. In all comparisons, most key drivers were unique to one network. The most overlaps were found between networks from the same dataset, followed by overlaps between the STARNET and GTEx versions of the same network. More dissimilar comparisons generally had fewer shared key drivers, and few key driver genes were shared between three or four networks (Fig S4A–D).

### Sample size differences partially explain differences in key driver prediction between male and female networks

We hypothesized that the increased predictive power of the male compared to the female networks could be because of the difference in input sample size. To test this, we randomly sub-sampled the male input genesets to include the same number of donors as the female networks, and constructed networks from these smaller sets. We identified an intermediate number of genes and edges for most sub-sampled networks (Table S7). There were also more shared key driver genes found in the sub-sampled male networks than between the full female networks. The predictions made by the sub-sampled networks recapitulated some of the full network predictions; both subcutaneous male sub-sampled networks (Table S7) recovered *ANAPC2* and *TYRO3* as key driver genes between the GTEx and STARNET versions. The smaller size and predictive power of the female networks appears to be only partially because of sample size differences.

Table S7 Connected genes, edges, and key drivers in full male networks (left), sub-sampled male networks (middle), female networks (right).

**Figure S5.**
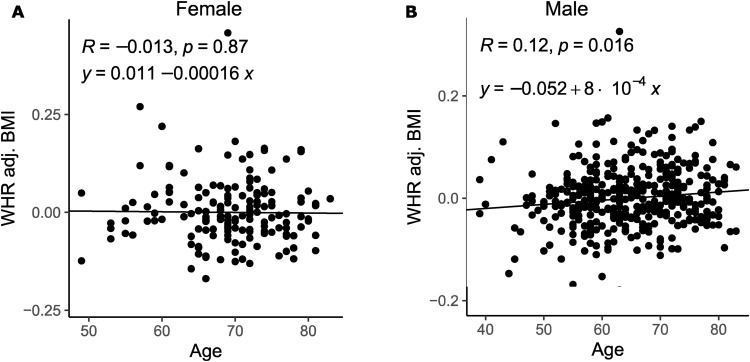
Correlation between age and WHR_adjBMI_ in Stockholm-Tartu Atherosclerosis Reverse Network Engineering Task (STARNET). **(A)** Pearson correlation between age and WHR_adjBMI_ in females in the STARNET cohort. **(B)** Pearson correlation between age and WHR_adjBMI_ in males in the STARNET cohort.

Because we observed that accounting for the number of donors could only partially correct the differences in predicted key drivers, we hypothesized that something intrinsic to the female donors/data may also contribute. We observe that the STARNET women are older than the GTEx women and may be entering menopause (Table 1). When comparing age to body fat distribution (WHR_adjBMI_), we found that STARNET men had a significant positive correlation, whereas the STARNET women’s age and WHR_adjBMI_ were not significantly correlated (Fig S5A and B).

**Figure S6.**
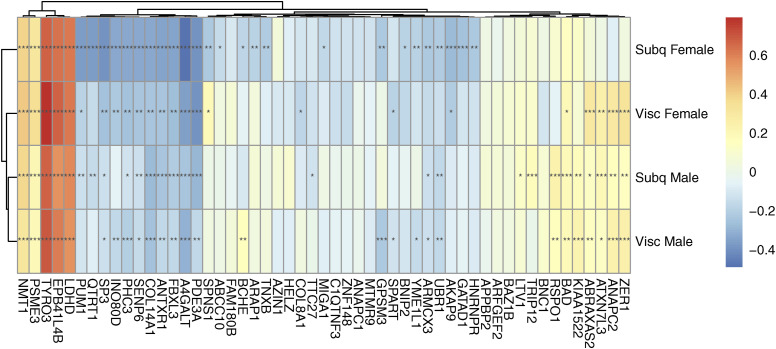
Correlation between KLF14 expression and the expression of the 53 key driver genes in Genotype-Tissue Expression project. Pearson correlations are shown by color, P-values adjusted using FDR correction shown with * (*** = adj.P < 0.001, * = adj.P < 0.05).

Finally, we know that transcription factor KLF14 is responsible for determining body fat distribution and related metabolic parameters in females (Small et al, 2018; Yang et al, 2022). We hypothesize that the differences in network structure may be because of regulation by KLF14. However, this gene was too lowly expressed in STARNET to be quantified and was not used to construct co-expression or Bayesian networks. In GTEx, the expression of *KLF14* was significantly correlated with many of the shared key driver genes, although only a handful have correlations specific to females (Fig S6). Within the 334 shared key driver genes identified, only *ACO1*, *ACAT1*, and *SLC25A1* are also part of the KLF14 trans-network.

### Prioritization pipeline identifies 53 novel and putatively functional adipocyte and pre-adipocyte key driver genes

**Figure 2.**
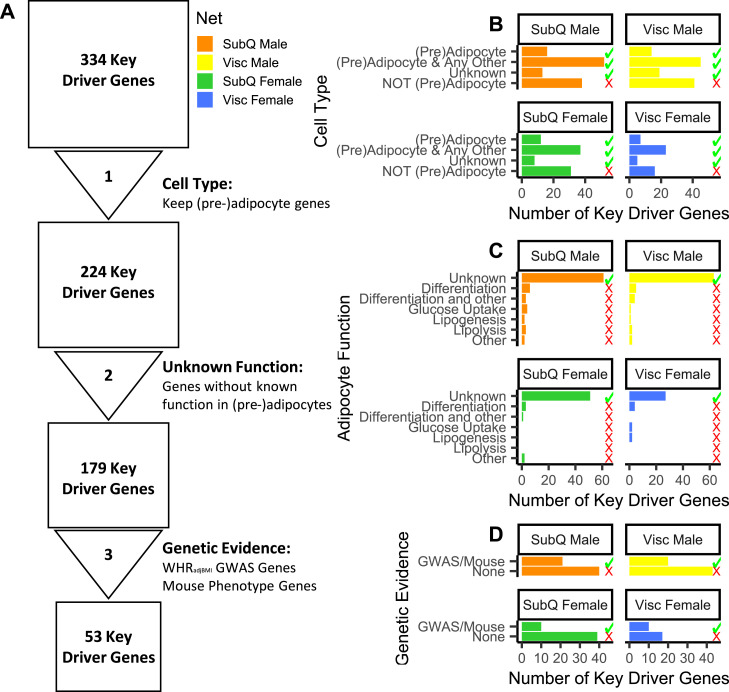
Prioritization of key driver genes for functional testing. **(A)** 334 key driver genes prioritized to 53 putative candidate regulators of WHR_adjBMI_ and adipocyte function using publicly available data. **(B)** Cell types in adipose tissue single cell and single nucleus RNA-seq data (Hepler et al, 2018; Min et al, 2019; Almanzar et al, 2020; Sun et al, 2020; Vijay et al, 2020; Hildreth et al, 2021; Sárvári et al, 2021) in which each key driver is expressed. **(C)** Key driver gene with known function in pre-adipocyte and adipocyte fat storage pathways. **(D)** Genetic evidence (status as WHR_adjBMI_ genome-wide association study gene or causes mouse fat storage phenotype) for key driver genes. Data Information: The green checkmark indicates genes kept in the analysis pipeline, and the red X indicates genes removed.

**Figure S7.**
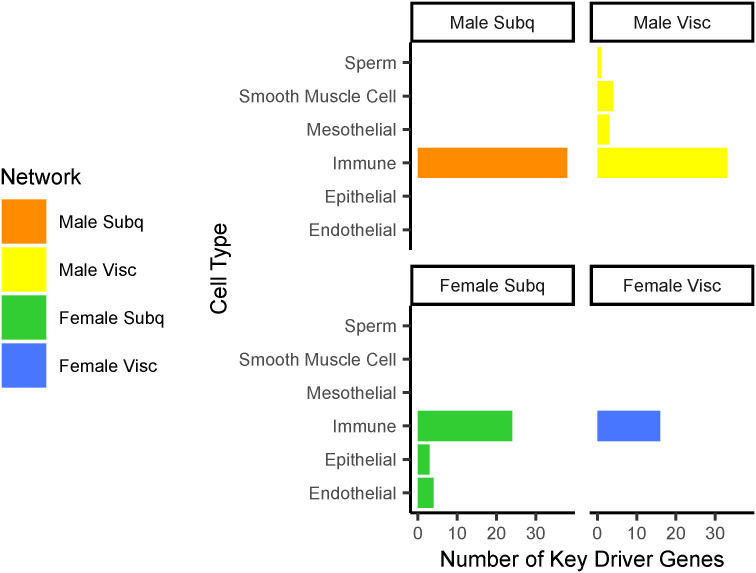
The 110 key driver genes removed from analyses because of primary expression in other cell types, in Fig 2, step 1.

**Figure S8.**
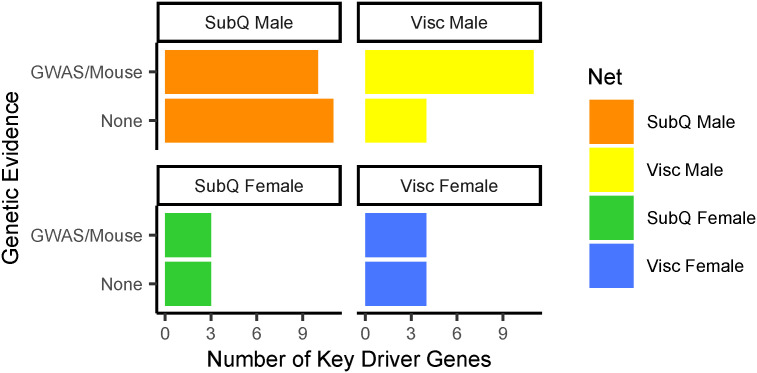
The 45 key driver genes with known function in adipose tissue that were removed in Fig 2, step 2 were subjected to the Genetic Evidence criteria used in Fig 2, step 3.

Because we hypothesize that fat distribution is driven, in large part, by pre-adipocyte and adipocyte expansion, we decided not to consider further any key driver genes that were primarily expressed in other cell types (see the Materials and Methods section, Fig 2A and B). Most of the 110 removed genes were expressed primarily in immune cells (Fig S7). To use the strength of Bayesian networks in proposing unbiased hypotheses, we characterized key driver genes that were previously unstudied in adipocytes. We performed a comprehensive search of the existing literature to identify genes with known function in adipocytes. As a result, we removed 45 key driver genes, including *FGF1*, *DPP4*, *LRP6*, and *RXRA*, involved in adipocyte processes (Fig 2A and C, Supplemental Data 1). To identify genes that likely regulate adipocyte fat storage, we prioritized 41 key driver genes nearby WHR_adjBMI_ GWAS loci and 15 key driver genes, when knocked out in mice, affect fat storage phenotypes (Fig 2A and D). In total, we prioritize 53 key driver genes with putative roles in human fat distribution via altered adipocyte fat storage that currently have unknown functions in adipose tissue (Table S6). Of the 45 well-studied key drivers, almost half were not near WHR_adjBMI_ GWAS loci, nor did they affect fat storage in mice (Fig S8). The Bayesian network and key driver identification approach was to identify these functional genes without traditional evidence.

Supplemental Data 1. Previously reported evidence of key driver genes regulating adipocyte fat storage.

### 23 prioritized key driver genes have roles in Wnt signaling and mitochondria in other cell types

**Figure S9.**
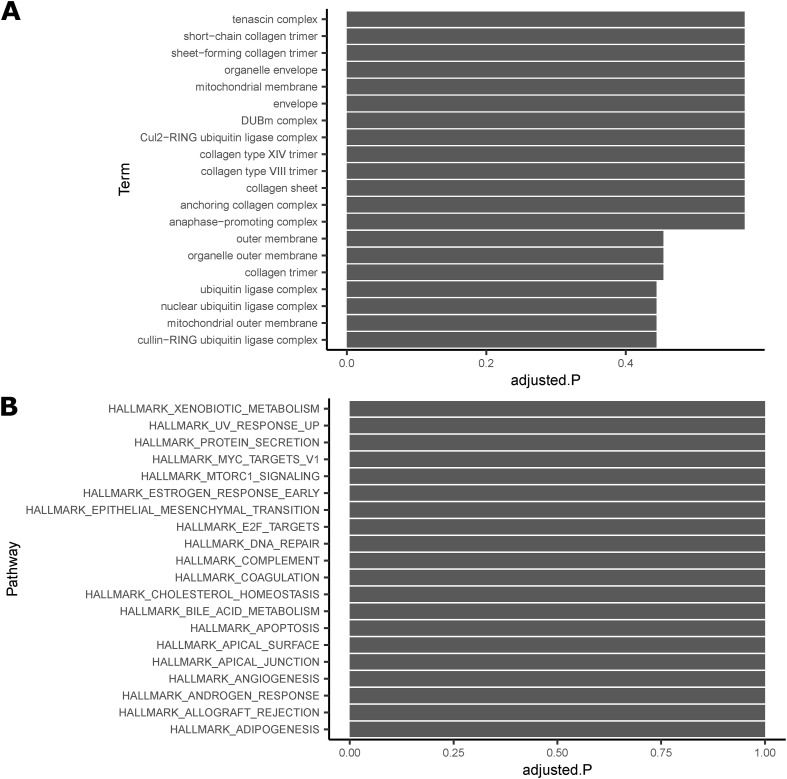
The 53 key driver genes prioritized for further study were not enriched for any specific pathways. **(A)** The top 20 Gene Ontology (GO) biological processes pathways, tanked by adjusted *P*-value. **(B)** The top 20 msigDB Hallmark pathways, ranked by *P*-value, adjusted *P*-value shown. Fisher’s exact test was used to test enrichments, FDR correction used to adjust *P*-values.

**Figure S10.**
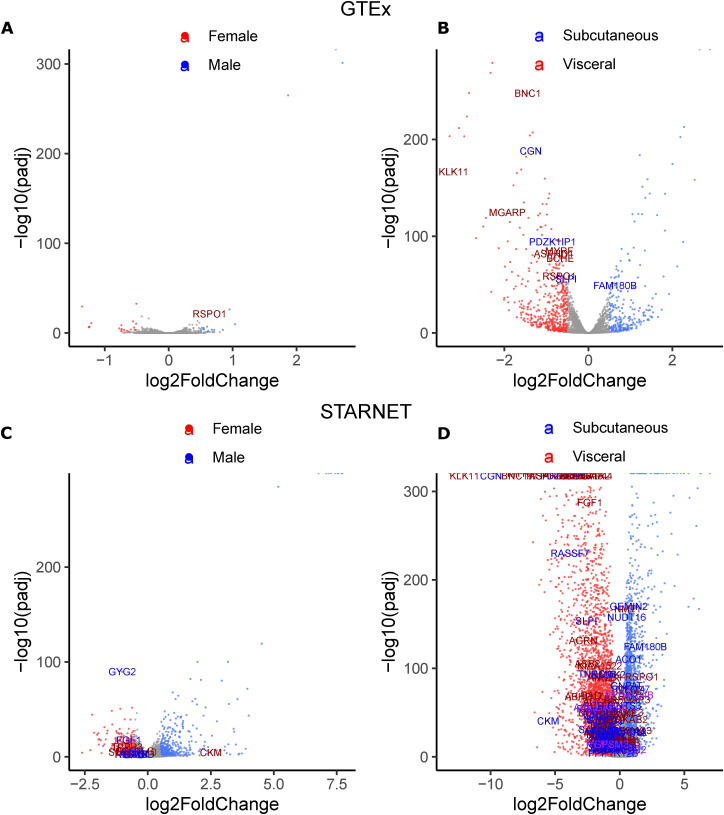
Differential expression of key driver genes in Genotype-Tissue Expression project (GTEx) and Stockholm-Tartu Atherosclerosis Reverse Network Engineering Task (STARNET). **(A)** Differential expression in GTEx between males and females. **(B)** Differential expression in GTEx between subcutaneous and visceral adipose tissue. **(C)** Differential expression in STARNET between males and females. **(D)** Differential expression in STARNET between subcutaneous and visceral adipose tissue Data Information: Differential expression performed with DESeq2 with Sex*Depot design matrix. Points show all expressed genes passing quality control, where the color denotes the sex or depot of significantly greater expression (fold change > 1, adjusted *P*-value < 0.05). Key driver genes with significant differential expression are labeled, where the color represents the sex or depot of the network in which it is a key driver. Purple labeled genes are key drivers in both networks.

**Figure S11.**
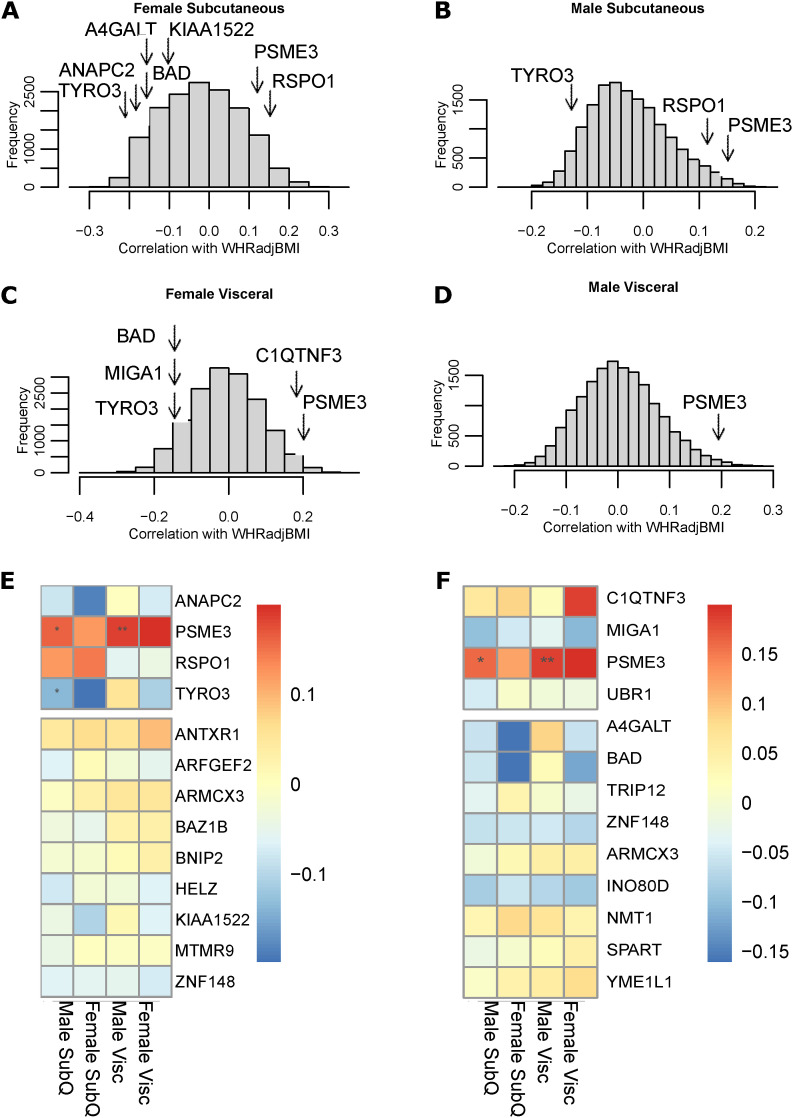
Gene expression correlations with WHR_adjBMI_ in Stockholm-Tartu Atherosclerosis Reverse Network Engineering Task (STARNET). **(A)** Correlation of WHR_adjBMI_ in STARNET with genes in female subcutaneous adipose. **(B)** Correlation of WHR_adjBMI_ in STARNET with genes in male subcutaneous adipose. **(C)** Correlation of WHR_adjBMI_ in STARNET with genes in female visceral adipose. **(D)** Correlation of WHR_adjBMI_ in STARNET with genes in male visceral adipose. **(E)** Correlation of Wnt key driver gene expression in adipose tissue with WHR_adjBMI_ in STARNET. **(F)** Correlation of mitochonria key driver gene expression in adipose tissue with WHR_adjBMI_ in STARNET. Data Information: Pearson correlations are shown by color, *P*-values adjusted using FDR correction shown with * (*** = adj.*P* < 0.001, * = adj.*P* < 0.05). In (A, B, C, D), arrows indicate prioritized Wnt-related genes (Table 3) and mitochondrial-related genes (Table 4) whose correlation with WHR_adjBMI_ is greater than 0.1.

To further characterize the 53 key driver genes, we attempted to find enrichment of specific pathway genes, but did not identify any enriched gene ontology (GO) terms or msigDB Hallmark pathways, likely because of the removal of well-characterized genes (Fig S9A and B). We performed a second literature search to determine the primary function of the 53 genes in other cell types and found that 13 affect the activity of the Wnt signaling pathway in other cell types (Table 3, Supplemental Data 2). These 13 genes were identified as key driver genes in all four sex-depot networks, and 10 are WHR_adjBMI_ GWAS candidate genes. We identified a different group of 13 genes that alter mitochondria function in other cell types, eight of which are WHR_adjBMI_ GWAS candidate genes (Table 4, Supplemental Data 3). Many key driver genes were differentially expressed between depots, whereas fewer were differentially expressed between sexes (Fig S10A–D). The expression of two key driver genes was significantly correlated with WHR_adjBMI_ (Fig S11A–F).

**Table 3.** Key driver genes that act in Wnt signaling in other cell types.

Key driver gene	Network		Public data	
	depot	sex	cell type	evidence
BAZ1B	Subcutaneous	Male	Multiple	GWAS
HELZ	Subcutaneous	Male	Multiple	GWAS
MTMR9	Subcutaneous	Male	Unknown	GWAS
TYRO3	Subcutaneous	Male	Unknown	GWAS
ANTXR1	Visceral	Male	Multiple	GWAS
ARFGEF2	Visceral	Male	Multiple	GWAS
ARMCX3	Visceral	Male	Multiple	Mouse
ANAPC2	Subcutaneous	Both	Adipocyte	GWAS
BNIP2	Subcutaneous	Both	Multiple	Mouse
KIAA1522	Visceral	Female	Unknown	GWAS
PSME3	Visceral	Female	Adipocyte	GWAS
RSPO1	Visceral	Female	Multiple	Mouse
ZNF148	Visceral	Female	Multiple	GWAS

Supplemental Data 2. Previously reported evidence of select key driver genes regulating Wnt signaling in non-adipocyte cell types.

**Table 4.** Key drivers that act in mitochondria in other cell types.

Key driver gene	Network		Public data	
	depot	sex	cell type	evidence
*INO80D*	Subcutaneous	Male	Adipocyte	Both
*SPART*	Subcutaneous	Male	Multiple	Mouse
*TRIP12*	Subcutaneous	Male	Multiple	GWAS
*A4GALT*	Visceral	Male	Adipocyte	GWAS
*ARMCX3*	Visceral	Male	Multiple	Mouse
*BAD*	Visceral	Male	Multiple	GWAS
*MIGA1*	Visceral	Male	Adipocyte	Mouse
*NMT1*	Visceral	Male	Multiple	GWAS
*YME1L1*	Both	Both	Multiple	Mouse
*C1QTNF3*	Visceral	Female	Adipocyte	GWAS
*PSME3*	Visceral	Female	Adipocyte	GWAS
*UBR1*	Visceral	Female	Multiple	Mouse
*ZNF148*	Visceral	Female	Multiple	GWAS

Supplemental Data 3. Previously reported evidence of select key driver genes regulating mitochondrial function in non-adipocyte cell types.

### Networks predict a role for four key driver genes in the Wnt signaling pathway

**Figure 3.**
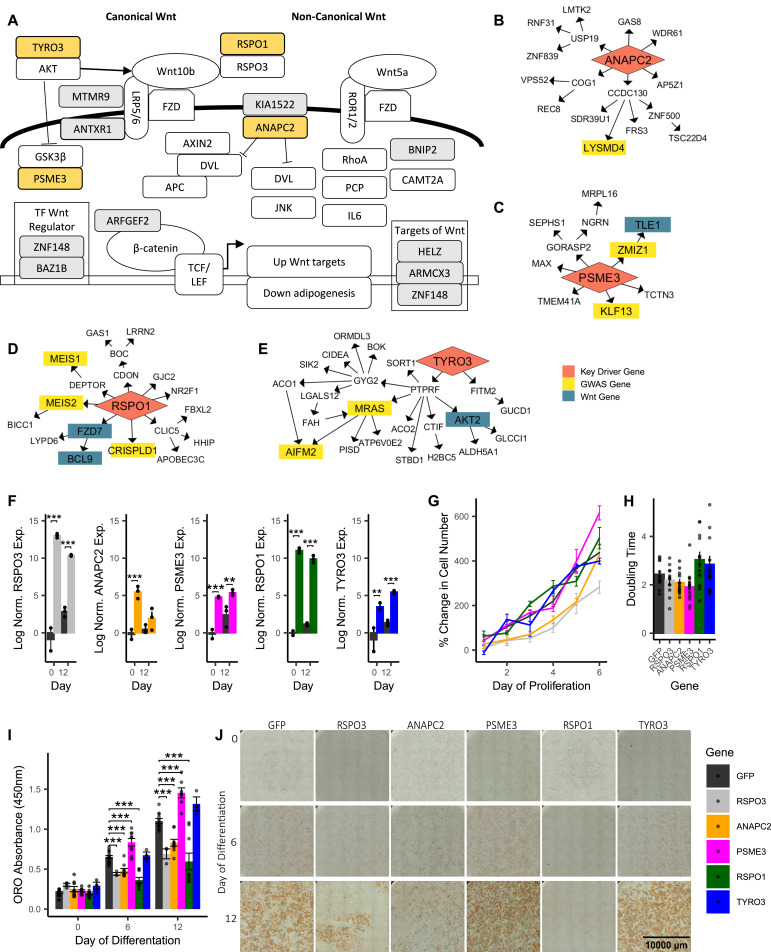
Prioritized key driver genes RSPO1, PSME3, and ANAPC2 affect fat storage in adipocytes. **(A)** The Wnt signaling pathway consists of canonical β-catenin signaling and non-canonical pathways. 13 key driver genes, shown in yellow and gray, interact with Wnt pathways in other cell types. **(B)** Key driver gene ANAPC2 in the Genotype-Tissue Expression project (GTEX) Subcutaneous Female network. **(C)** Key driver gene PSME3 in the Stockholm-Tartu Atherosclerosis Reverse Network Engineering Task Visceral Female network. **(D)** Key driver gene RSPO1 in the GTEx Visceral Female network. **(E)** Key driver gene TYRO3 in the GTEx Subcutaneous Male network. **(F)** Expression of key driver genes compared with GFP controls on day 0 and 12 after onset of differentiation (n = 3). **(G)** Percent change in cell number over 6 d (n = 4). **(H)** Calculated doubling time in days (n = 12). **(I, J)** Quantification of Oil Red O staining of cells, performed for each gene of interest and GFP controls on day 0, 6 and 12 after beginning differentiation, (J) representative images of one well of a 12-well plate are shown (n = 3–15). Data Information: (B, C, D, E) Four selected key driver genes (red) regulate both WHR_adjBMI_ downstream genes (Pulit et al, 2019) (yellow) and Wnt signaling downstream genes (blue, GO term “Wnt signaling pathway”) in GTEx and Stockholm-Tartu Atherosclerosis Reverse Network Engineering Task. **(F, G, H, I, J)** RSPO3 serves as a positive control. All plots show mean ± SEM. Differences between groups were determined using two-way ANOVA by day and gene (Gene of Interest versus GFP controls), and post hoc tests were performed using pooled *t* test with Dunnett’s adjustment. Adjusted *P*-values shown with * (*** = adj.*P* < 0.001, ** = adj.*P* < 0.01, * = adj.*P* < 0.05).

**Figure S12.**
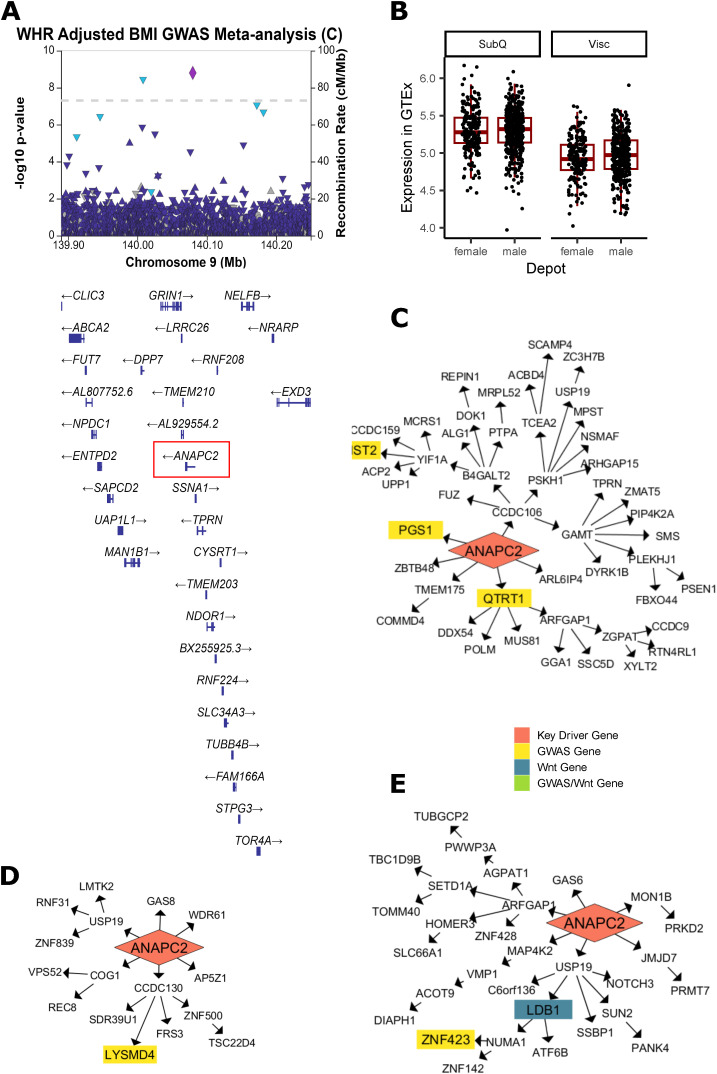
Additional evidence of ANAPC2 involvement in WHR_adjBMI_ and Wnt signaling. **(A)** A significant genome-wide association study (GWAS) signal is detected near ANAPC2 in WHR_adjBMI_ GWAS meta-analysis (Pulit et al, 2019). **(B)** In Genotype-Tissue Expression project, both males and females show higher expression of ANAPC2 in subcutaneous fat depots over visceral depots, although the change is non-significant after *P*-value adjustment. **(C, D, E)** ANAPC2 is a key driver that regulates downstream WHR_adjBMI_ GWAS genes in the Stockholm-Tartu Atherosclerosis Reverse Network Engineering Task subcutaneous male network, (D) the Genotype-Tissue Expression project subcutaneous male network, and (E) regulates both WHR_adjBMI_ GWAS genes and Wnt genes in Stockholm-Tartu Atherosclerosis Reverse Network Engineering Task female subcutaneous.

**Figure S13.**
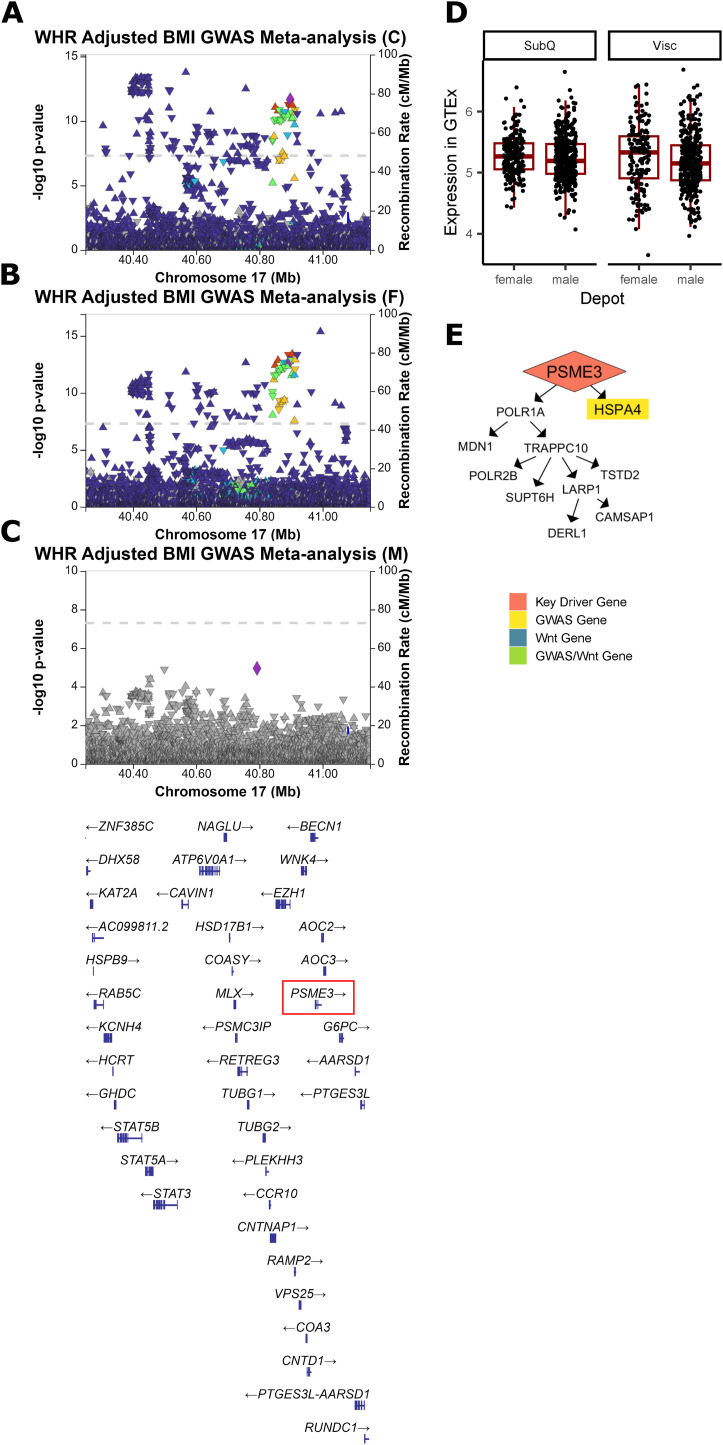
Additional evidence of PSME3 involvement in WHR_adjBMI_. **(A)** A significant genome-wide association study (GWAS) signal is detected near PSME3 in WHR_adjBMI_ GWAS meta-analysis (Pulit et al, 2019). **(B, C)** This same signal is strongly associated with WHR_adjBMI_ in the female-specific GWAS meta-analysis, (C) but is not present in the male-specific GWAS. **(D)** In Genotype-Tissue Expression project, there are no significant changes in PSME3 gene expression between depots or sexes. **(E)** PSME3 regulates WHR_adjBMI_ GWAS gene in the Genotype-Tissue Expression project Visceral Female network.

**Figure S14.**
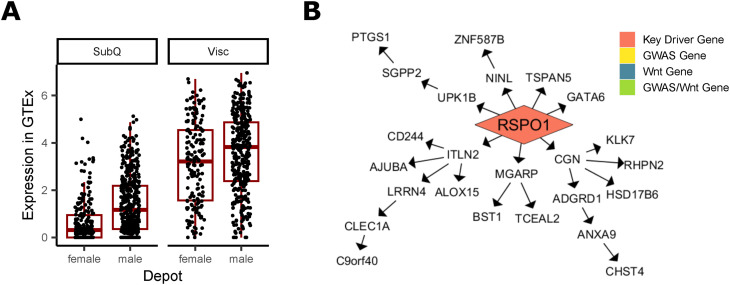
Additional evidence of RSPO1 involvement in WHR_adjBMI_. **(A)** In Genotype-Tissue Expression project, both sexes show higher expression of RSPO1 in visceral fat depots over subcutaneous depots (adj.*P* = 5.1 × 10^−21^), and males show higher expression than females in both depots (adj.*P* = 4.9 × 10^−57^). **(B)** RSPO1 is a key driver that regulates many downstream genes in the Stockholm-Tartu Atherosclerosis Reverse Network Engineering Task female visceral network.

**Figure S15.**
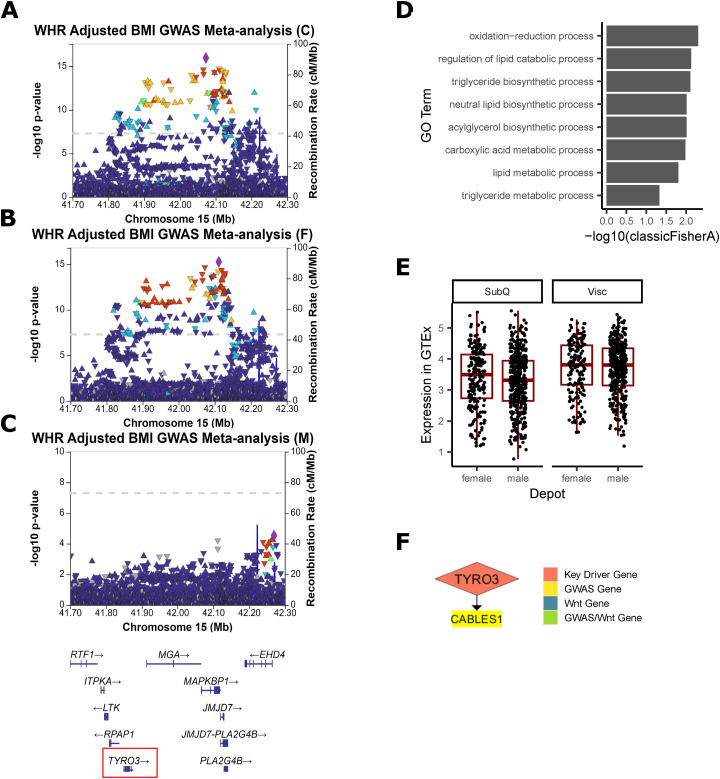
Additional evidence of TYRO3 involvement in WHR_adjBMI_. **(A)** A significant genome-wide association study (GWAS) signal is detected near TYRO3 in WHR_adjBMI_ GWAS meta-analysis (Pulit et al, 2019). **(B, C)** This same signal is strongly associated with WHR_adjBMI_ in the female-specific GWAS meta-analysis, (C) but is not present in the male-specific GWAS. **(D)** In Genotype-Tissue Expression project subcutaneous male networks, the genes downstream of TYRO3 are significantly enriched for lipid-related biological processes. **(E)** In Genotype-Tissue Expression project, both males and females show higher expression of TYRO3 in visceral fat depots over subcutaneous depots, although the change is non-significant after *P*-value adjustment. **(F)** TYRO3 regulates one WHR_adjBMI_ GWAS gene in the Stockholm-Tartu Atherosclerosis Reverse Network Engineering Task subcutaneous male network.

Because canonical Wnt signaling can transcriptionally repress adipogenic genes *PPARɣ* and *CEBPα* (Teo & Kahn, 2010; Chen & Wang, 2018) (Fig 3A), we chose four of the 13 Wnt-related key driver genes that act upstream of β-catenin and TCF/LEF in other cell types. PSME3 (proteasome activator subunit 3, REG-ɣ, PA28-ɣ) (Li et al, 2015; Chen et al, 2017), RSPO1 (r-spondin 1) (Binnerts et al, 2007; Gore et al, 2011), and TYRO3 (TYRO3 protein tyrosine kinase) (Zhu et al, 2016; Chen et al, 2020) were previously shown to activate Wnt, while ANAPC2 (anaphase-promoting complex 2) inhibits Wnt signaling (Ganner et al, 2009). Their role in adipose remains unknown, although multiple lines of evidence suggest they may have a regulatory role in fat storage (Figs 3B–E, S12A–E, S13A–E, S14A and B, and S15A–E).

In networks, these four genes regulate a large number of downstream genes (Figs 3B–E, S12C–E, S13E, S14B, and S15F), including WHR_adjBMI_ GWAS candidate genes (Table S6), and canonical Wnt signaling pathway genes. *TYRO3*’s downstream genes are significantly enriched for lipid-related gene ontology (GO) terms (Fig S15D).

**Figure S16.**
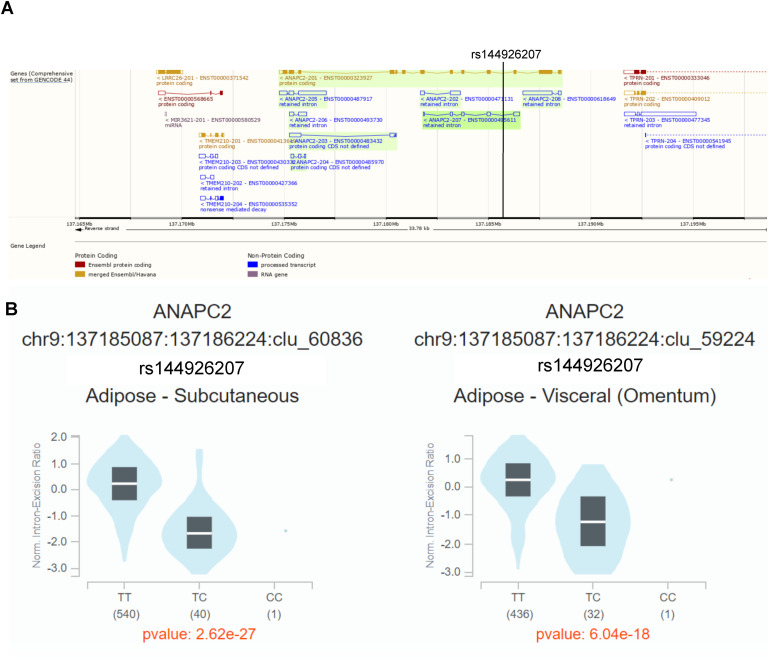
Genetic regulation of ANAPC2 intronic excision by WHR_adjBMI_ single nucleotide polymorphism rs144926297 in Genotype-Tissue Expression project. **(A)** Alternate transcripts of ANAPC2 in GENCODE 44, the lead Single nucleotide polymorphism in the ANAPC2 WHR_adjBMI_, rs144926297 is located within the intron. **(B)** Normalized intronic excision by genotype in subcutaneous and visceral fat in Genotype-Tissue Expression project.

*ANAPC2*, *PSME3*, and *TYRO3* are located within the gene dense WHR_adjBMI_ GWAS loci, and may be the causal gene at the locus. The signals near *PSME3* and *TYRO3* are both specific to females (Figs S13A–C and S15A–C). The lead single nucleotide polymorphism (SNP) in the *ANAPC2* locus, rs144926207, is located in the intronic region of *ANAPC2*, and reference allele T is associated with higher WHR_adjBMI_ (Pulit et al, 2019, Fig S12A) and higher excision of an intronic region of *ANAPC2* in subcutaneous and visceral depots in GTEx (Fig S16A and B). *RSPO1* shows strong differential expression between sexes and between depots (Fig S14A). All four genes had strong correlations with WHR_adjBMI_ in STARNET (Fig S11A–D) and these correlations differ by sex or by depot. By virtue of their status as prioritized network key driver genes, we hypothesize that these four genes affect fat storage in adipocytes, potentially through canonical Wnt signaling activity.

#### *ANAPC2*, *PSME3*, and *RSPO1* overexpression alter adipogenesis, not proliferation

To test these hypotheses, we overexpressed each of the four Wnt key driver genes or a GFP control plasmid in a human male pre-adipocyte cell line (Wabitsch et al, 2001; Fischer-Posovszky et al, 2008) using lenti-virus (see the Materials and Methods section, Fig 1). We confirmed the overexpression of each gene compared to GFP controls using qRT-PCR (Fig 3F). We also overexpressed gene *RSPO3* as a positive control because previous literature shows that *RSPO3* does not affect subcutaneous pre-adipocyte proliferation but impairs adipogenesis (Loh et al, 2020).

We assessed the ability of these cell lines to proliferate by seeding each at the same density and counting representative wells every 24 h (see the Materials and Methods section). We found no differences in the rate of increase in cell number or the mean doubling time during exponential growth between any cell lines and GFP controls (Fig 3G and H).

**Figure S17.**
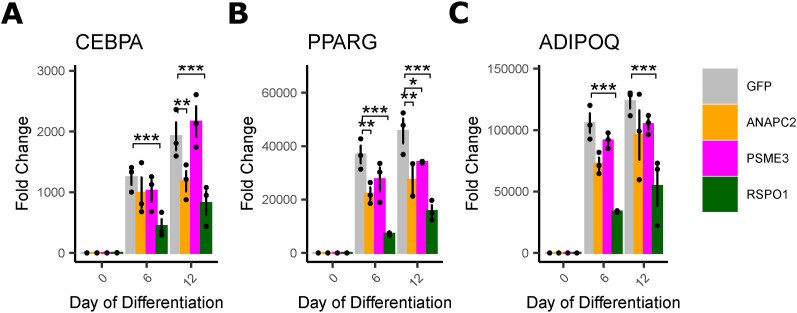
Markers of adipogenesis increase over time in differentiating per-adipocyte. **(A)** Expression of CEBPA in cells overexpressing genes of interest. **(B)** Expression of PPARG in cells overexpressing genes of interest. **(C)** Expression of ADIPOQ in cells overexpressing genes of interest. Data Information: n = 3 replicates used in all assays. Differences between groups were determined using one-way ANOVA within each timepoint by gene (Gene of Interest versus GFP controls). All post hoc tests were performed using pooled *t* test with Dunnett’s adjustment. Adjusted *P*-values shown with * (*** = adj.*P* < 0.001,* = adj.*P* < 0.05, ^#^ = adj.*P* < 0.1).

We quantified the alterations in adipogenesis because of key driver overexpression by staining cells with the neutral lipid specific dye, Oil Red O (ORO), at 0, 6, and 12 d after the onset of differentiation. As expected, we observed an increase in lipid accumulation on day 6 and 12 compared with day 0 in all cell lines, confirming successful induction of adipogenesis, with a 4.25-fold increase in ORO absorbance in GFP controls on day 12 compared with day 0 (Fig 3I and J). Compared with GFP controls, *ANAPC2* and *RSPO1* overexpressing cells were deficient in adipogenesis, with 24.6% and 45.4% less lipid accumulation, respectively, than GFP controls on day 12. We observed that *PSME3* overexpressing cells had a 31.6% increase in lipid accumulation compared with controls on day 12. *TYRO3* overexpression caused no significant differences in lipid accumulation from controls at any time point. Positive control *RSPO3* overexpressing cells were also deficient in lipid accumulation by 37.3% compared with controls on day 12, consistent with previous studies (Loh et al, 2020). Expression of adipocyte markers *CEBPα*, *PPARɣ*, and *ADIPOQ* also increased over 12 d, and showed significant inhibition in *ANAPC2* and *RSPO1* overexpressing cells compared with controls, in agreement with adipogenesis (Fig S17A–C).

To fully characterize the effects of these genes, we would need to perform the same functional studies in both subcutaneous and visceral cell lines to determine the magnitude of adipogenic alterations in each cell type and infer effects on body fat distribution. In the absence of these data, we used the correlations with WHR_adjBMI_ in STARNET (Fig S11E) to determine if our data could explain the relationship between gene expression and body fat distribution. *RSPO1* findings are well aligned with the human data—e.g., if RSPO1 inhibits adipocyte fat storage in both subcutaneous (Fig 3D) and visceral adipose tissue, then expression of *RSPO1* in visceral adipose tissue should decrease visceral fat storage and hence decrease WHR_adjBMI_, and expression in subcutaneous adipose tissue should decrease subcutaneous fat storage to increase WHR_adjBMI_. This is perfectly replicated in STARNET correlation data (Fig S11E)—we observe positive correlations between subcutaneous *RSPO1* expression and WHR_adjBMI_, but we observe negative correlations between visceral *RSPO1* expression and WHR_adjBMI_. Although *t*he correlation data do not immediately explain *ANAPC2* and *PSME3* findings, phenotypic effects in visceral adipocytes of different magnitudes or directions could explain the observed changes.

#### *RSPO1* activates *Wnt* signaling to inhibit adipogenesis

**Figure 4.**
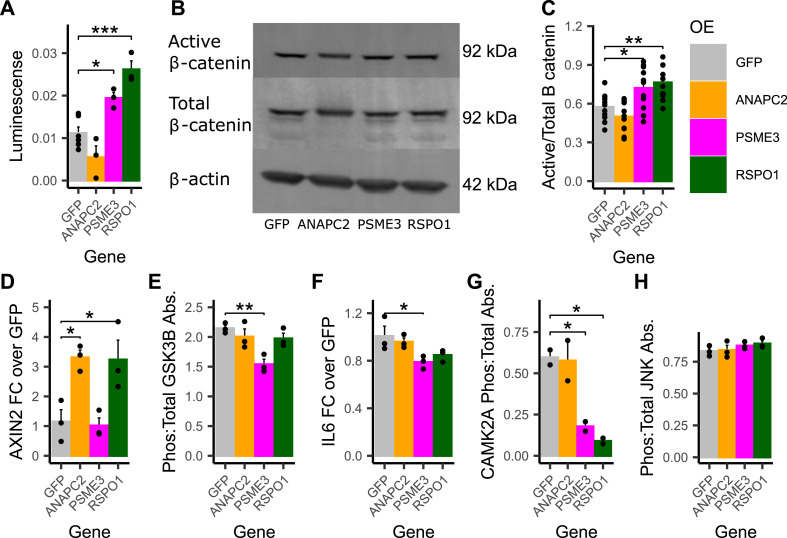
RSPO1 and PSME3 activate canonical Wnt signaling when inhibiting the Ca^2+^ non-canonical Wnt pathway. **(A)** Wnt transcriptional activity measured by luminescence of luciferase reporter (n = 3–6). **(B, C)** Representative images and (C) Quantification of active (non-phosphorylated) and total β-catenin by immunoblotting (n = 12). **(D)** Gene expression of AXIN2 measured by qRT-PCR (n = 3). **(E)** Ratio of active (phosphorylated): total GSK3β measured by ELISA (n = 3). **(F)** Gene expression of IL6 measured by qRT-PCR (n = 3). **(G)** Ratio of active (phosphorylated): total CAMK2A measured by ELISA (n = 2). **(H)** Ratio of active (phosphorylated): total JNK measured by ELISA (n = 3). Data Information: All plots show mean ± SEM. Differences between groups were determined using one-way ANOVA by gene (Gene of Interest versus GFP controls), and post hoc tests were performed using pooled *t* tests with Dunnett’s adjustment. Adjusted *P*-values shown with * (*** = adj.*P* < 0.001, ** = adj.*P* < 0.01, * = adj.*P* < 0.05).

We assessed the activity of the canonical Wnt signaling pathway using cells expressing both an overexpression plasmid and the 7TFC-luciferase construct, which measures the output of β-catenin-TCF/LEF transcription via luminescence (see the Materials and Methods section). *PSME3* and *RSPO1* overexpressing cells were able to activate canonical Wnt signaling significantly more than GFP controls; luminescence increased by 0.74-fold and 1.34-fold in *PSME3* and *RSPO1* overexpressing cells, respectively (Fig 4A).

Next, we looked at individual molecules of the canonical Wnt signaling pathway (Chen & Wang, 2018). We quantified the ratio of active (non-phosphorylated) β-catenin to total β-catenin using immunoblotting (Fig 4B and C), and we observed that *PSME3* and *RSPO1* overexpressing cells had a 25.4% and 33.1% increase in active/total β-catenin species compared with controls, respectively, consistent with the luciferase reporter assay. We quantified *AXIN2* mRNA expression, a target of canonical Wnt signaling, using qRT-PCR (Fig 4D). We observed that, compared to GFP control cells, cells overexpressing *ANAPC2* and *RSPO1* increased *AXIN2* expression by 1.9-fold and 2.2-fold, respectively. Finally, using ELISAs, we measured the ratio of active to total GSK3β, an inhibitor of the canonical Wnt signaling pathway (Fig 4E). Compared with GFP controls, we observed a 28.2% decrease in the ratio of active to total GSK3β in *PSME3* overexpressing cells.

We also assessed the consequences of gene overexpression on non-canonical Wnt signaling (Ackers & Malgor, 2018; Akoumianakis et al, 2022). We observed a significant 21.7% decrease in the mRNA expression of *IL6*, a target of multiple types of non-canonical Wnt signaling, in *PSME3* overexpressing cells (Fig 4F). *RSPO1* overexpressing cells also show a non-significant 15.8% decrease in *IL6* expression. Using ELISAs, we measured the ratio of active to total CAMK2A, a member of the Ca^2+^ non-canonical Wnt pathway, and we observed a significant 70.2% and 85.0% decrease in active to total CAMK2A ratio in *PSME3* and *RSPO1* overexpressing cells, respectively (Fig 4G). Finally, we measured the ratio of active to total JNK, a member of the planar cell polarity non-canonical Wnt pathway. We observed no differences between overexpressing cell lines and GFP controls (Fig 4H).

### Networks predict a role for four key driver genes related to mitochondrial function

**Figure 5.**
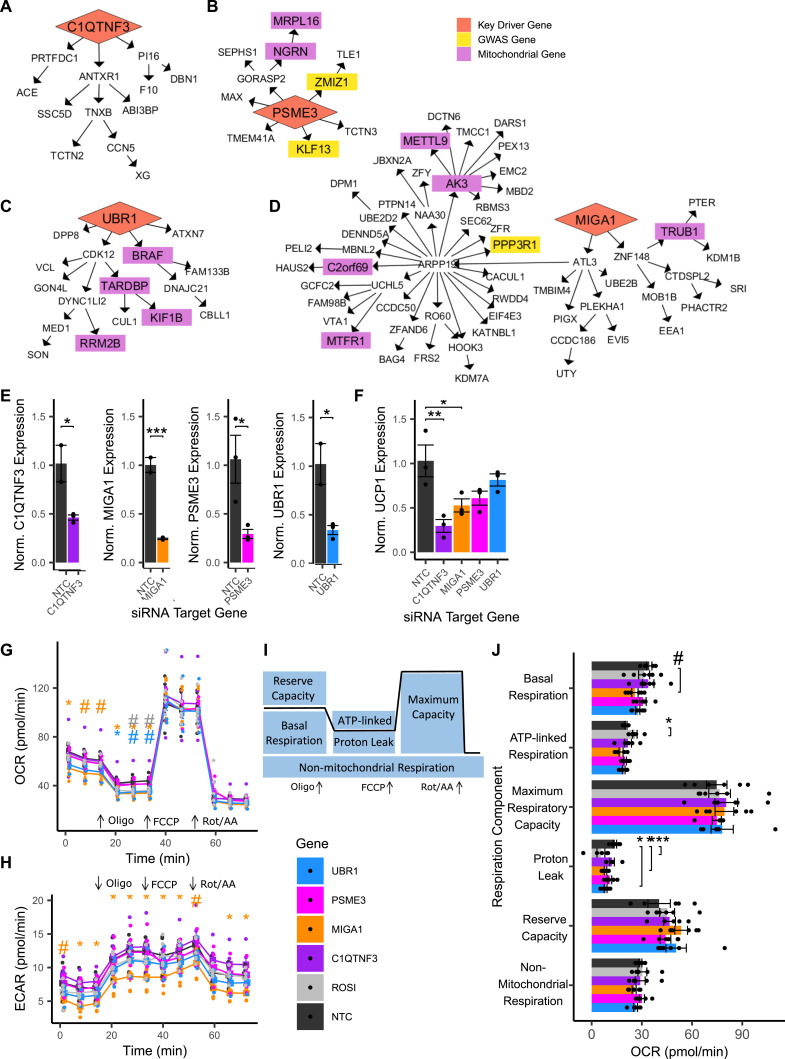
Prioritized key driver genes MIGA1 and UBR1 affect mitochondrial function in adipocytes. **(A)** Key driver gene C1QTNF3 in the Genotype-Tissue Expression project (GTEx) Visceral Female network. **(B)** Key driver gene UBR1 in the GTEx Visceral Female network. **(C)** Key driver gene MIGA1 in the GTEx Visceral Male network. **(D)** Key driver gene PSME3 in the Stockholm-Tartu Atherosclerosis Reverse Network Engineering Task Visceral Female network. **(E)** Gene expression of key driver genes in non-targeting control (NTC) cells and in siRNA knockdown lines. **(F)** Expression of UCP1 in siRNA knockdown lines and controls. **(G)** Oxygen consumption rates genes in NTC cells and in siRNA knockdown lines. **(H)** Extracellular acidification rates genes in NTC cells and in siRNA knockdown lines. **(I)** Phenotypes calculated from oxygen consumption rates under various stimulations. **(J)** Analysis of mitochondrial phenotypes upon siRNA perturbation under stimulations. Data Information: (A, B, C, D) Four selected key driver genes are regulated both WHR_adjBMI_ downstream genes (Pulit et al, 2019) (yellow) and mitochondrial downstream genes (purple, GO term “Mitochondrion”) in GTEx and Stockholm-Tartu Atherosclerosis Reverse Network Engineering Task. **(E, F, G, H, I, J)** n = 6 replicates used in all assays. ROSI cells were treated with 2 μM rosiglitazone for 24 h before assay. **(E, F, I)** Differences between groups in (E, F, I) were determined using one-way ANOVA by gene (Gene of Interest versus NTC). **(G, H)** Differences between groups in (G, H) were determined using one-way ANOVA within each timepoint by gene (Gene of Interest versus NTC). All post hoc tests were performed using pooled *t* test with Dunnett’s adjustment. Adjusted *P*-values shown with * (*** = adj.*P* < 0.001,* = adj.*P* < 0.05, ^#^ = adj.*P* < 0.1).

**Figure S18.**
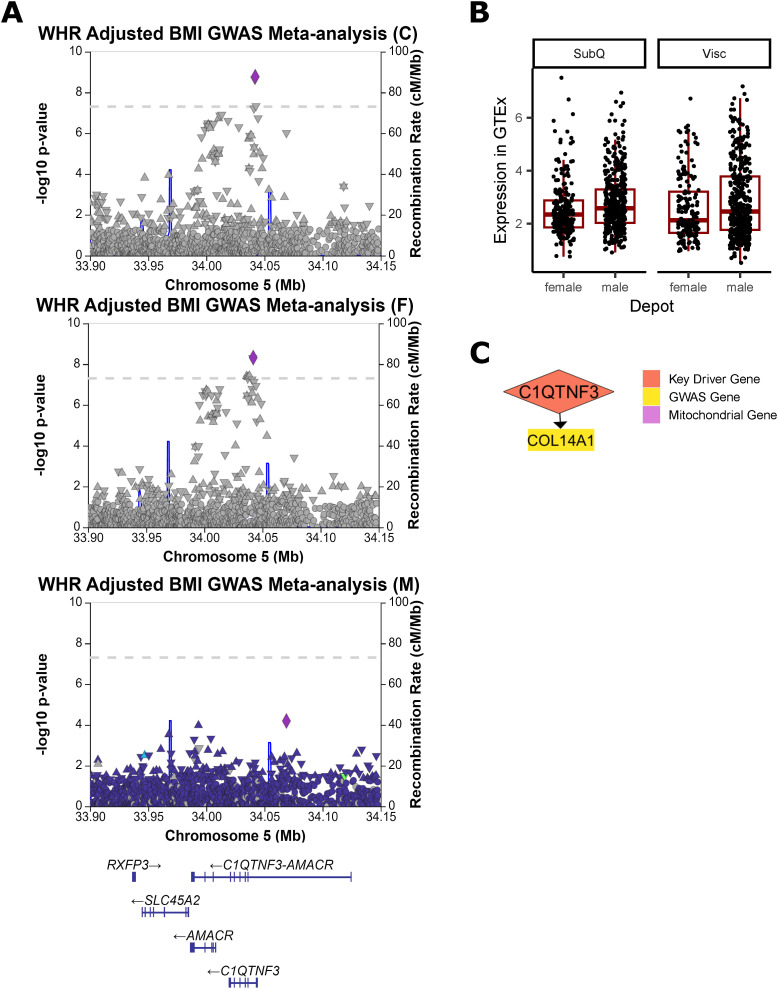
Additional evidence of C1QTNF3 involvement in WHR_adjBMI_. **(A)** A significant genome-wide association study (GWAS) signal was detected near C1QTNF3 in WHR_adjBMI_ GWAS meta-analysis (Pulit et al, 2019). This same signal is strongly associated with WHR_adjBMI_ in the female-specific GWAS meta-analysis, but is not present in the male-specific GWAS. **(B)** In Genotype-Tissue Expression project, both males and females show higher expression of C1QTNF3 in visceral fat depots over subcutaneous depots, although the change is non-significant after *P*-value adjustment. **(C)** C1QTNF3 regulates one WHR_adjBMI_ GWAS gene in the Stockholm-Tartu Atherosclerosis Reverse Network Engineering Task subcutaneous male network.

**Figure S19.**
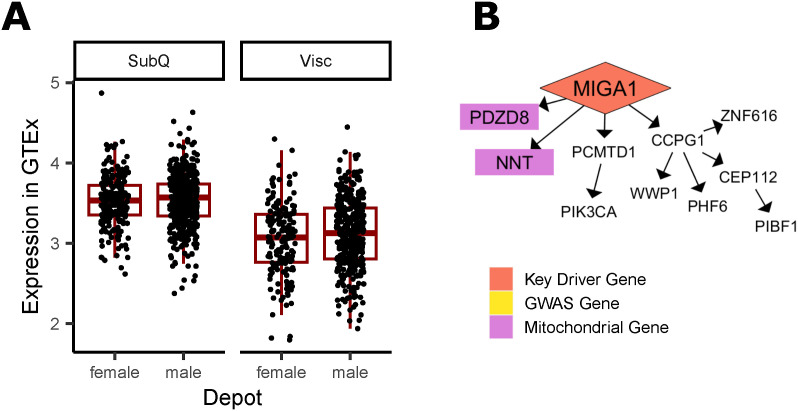
Additional evidence of MIGA1 involvement in WHR_adjBMI_. **(A)** In Genotype-Tissue Expression project, both males and females show higher expression of MIGA1 in visceral fat depots over subcutaneous depots, although the change is non-significant after *P*-value adjustment. **(B)** MIGA1 regulates mitochondrial genes in the Stockholm-Tartu Atherosclerosis Reverse Network Engineering Task subcutaneous male network.

**Figure S20.**
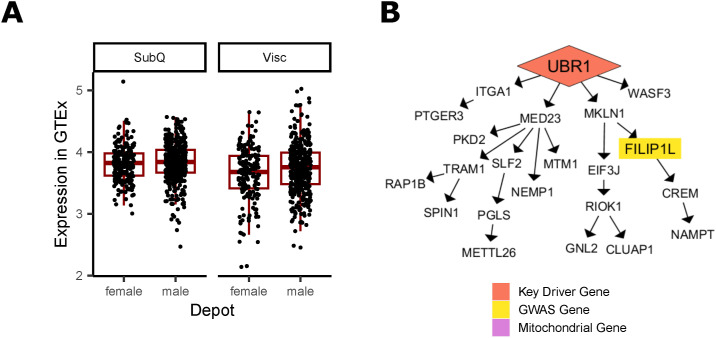
Additional evidence of UBR1 involvement in WHR_adjBMI_. **(A)** In Genotype-Tissue Expression project, both males and females show higher expression of UBR1 in visceral fat depots over subcutaneous depots, although the change is non-significant after *P*-value adjustment. **(B)** UBR1 regulates one WHR_adjBMI_ genome-wide association study genes in the Stockholm-Tartu Atherosclerosis Reverse Network Engineering Task subcutaneous male network.

Because mitochondria can impact adipocyte function and lipid storage in various ways, we chose four of the 13 mitochondrial-related key driver genes (Table 4) with diverse functions to study further. In other cell types, C1QTNF3 (Complement C1q Tumor Necrosis Factor-Related Protein 3) increases mitochondrial biogenesis, oxygen consumption, and ATP synthesis (Feng et al, 2016; Zhang et al, 2017; Gao et al, 2020), and *MIGA1*’s protein (Mitoguardin 1) promotes mitochondrial fusion (Liu et al, 2016; Zhang et al, 2016). *Psme3* is anti-apoptotic and promotes mitochondrial health in mice (Moncsek et al, 2015; Xie et al, 2020), and *UBR1* (Ubiquitin Protein Ligase E3 Component N-Recognin 1) targets misfolded mitochondrial proteins (Tran, 2019; Metzger et al, 2020) and prevents mitophagy (Yamano & Youle, 2013). Their role in adipocytes remains unknown, although multiple lines of evidence suggest they may have a regulatory role in fat storage (Figs 5A–D, S13A–E, S18A–C, S19A and B, and S20A and B).

**Figure S21.**
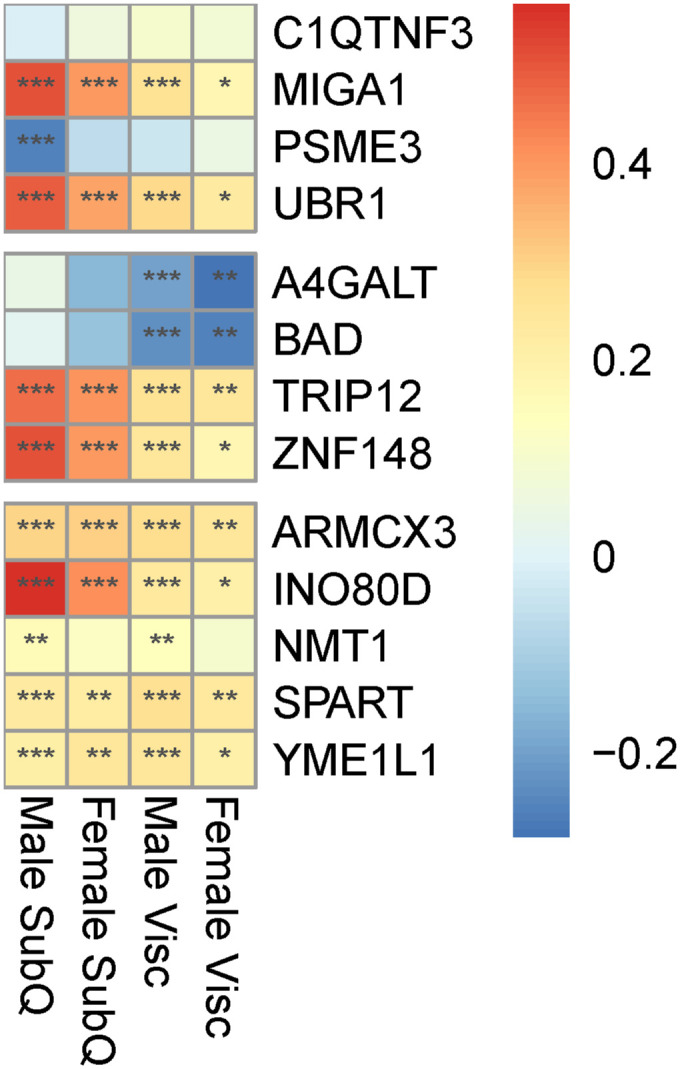
Correlation of UCP1 expression in adipose tissue with WHR_adjBMI_ in Stockholm-Tartu Atherosclerosis Reverse Network Engineering Task. Data Information: Pearson correlations are shown by color, *P*-values adjusted using FDR correction shown with * (*** = adj.*P* < 0.001, * = adj.*P* < 0.05).

In networks, these four genes regulate a large number of downstream genes (Figs 5A–D, S13E, S18C, S19B, and S20B), including WHR_adjBMI_ GWAS candidate genes (Table S6) and genes related to mitochondrial function. We find that *C1QTNF3* and *PSME3* are found near WHR_adjBMI_ GWAS loci along with other genes, and we hypothesize they may be the causal gene in these loci, whereas *MIGA1* and *UBR1* are putative mechanistic genes that are not in GWAS loci. Confirming their likely role in adipocyte mitochondria, we find that the expression of these genes is significantly correlated to *UCP1* expression in adipose tissue (Fig S21).

Because these four genes are prioritized network key driver genes, we hypothesize that they affect mitochondrial function in adipocytes (Fig 1).

#### Knockdown of MIGA1 and UBR1 inhibits oxygen consumption in differentiated adipocytes

To test these hypotheses, we down-regulated each of the four genes using siRNA in primary human female pre-adipocyte cells, with non-targeting siRNA as a control (see the Materials and Methods section). We obtained mature adipocytes by differentiating these cells for 18 d, then measured the mRNA expression of each gene to confirm that the knockdown efficiency was still more than 50% compared to controls (Fig 5E).

**Figure S22.**
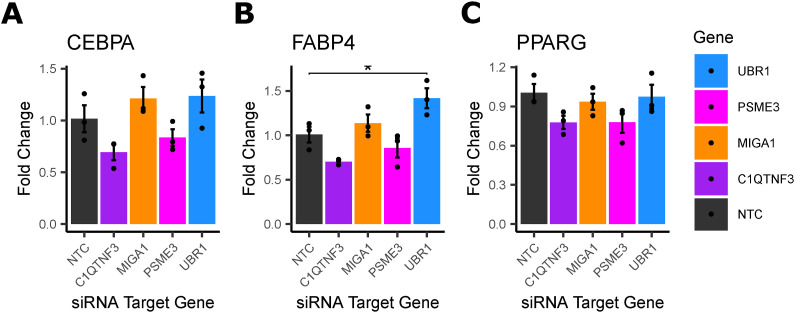
Expression of mature adipocyte marker genes in differentiated primary cells. **(A)** Gene expression of CEBPA, in mature adipocytes treated with siRNA for the indicated gene. **(B)** Gene expression of PPARG, in mature adipocytes treated with siRNA for the indicated gene. **(C)** Gene expression of FAPB4 in mature adipocytes treated with siRNA for the indicated gene. Data Information: n = 3–6 replicates used in all assays. Differences between groups were determined using one-way ANOVA by gene (Gene of Interest versus non-targeting controls). Post hoc tests were performed using pooled *t* test with Dunnett’s adjustment. Adjusted *P*-values shown with * (*** = adj.*P* < 0.001,* = adj.*P* < 0.05, ^#^ = adj.*P* < 0.1).

We examined the effect of the knockdown of the remaining four genes on *UCP1* expression in differentiated adipocytes. We found that *C1QTNF3* and *MIGA1* knockdown significantly reduced *UCP1* expression, 71.1%, and 48.7%, respectively, compared with controls (Fig 5F). We also measured the expression of mature adipocyte markers, *PPARG*, *CEPBA*, and *FAPB4*. *UBR1* knockdown resulted in a significant 40.6% increase in *FABP4* expression (Fig S22A–C).

We then determined the effect of gene knockdown on cellular oxygen consumption rate (OCR) using the Seahorse assay (Ding et al, 2018) (see the Materials and Methods section). Importantly, we found that *MIGA1* knockdown significantly reduced the basal OCR and OCR after adding ATP synthase inhibitor compared with controls (Fig 5G). In addition, we found that *UBR1* knockdown significantly reduced the OCR after adding ATP synthase inhibitor compared with controls. Only *MIGA1* knockdown resulted in significantly reduced extracellular acidification rate (ECAR) in differentiated adipocytes compared with controls (Fig 5H).

Because each stimulation used in the OCR assay inhibits specific parts of the respiratory chain, we derived deeper mitochondrial phenotypes (Rose et al, 2014; Son et al, 2017) (Fig 5I, see the Materials and Methods section). For example, we calculated the amount of oxygen consumed by mitochondrial proton leak, which refers to the futile H^+^ shuttling across the outer mitochondrial membrane that does not produce ATP. Thermogenic uncoupling through UCP1 contributes to proton leak (Bertholet et al, 2022). Both *MIGA1* and *UBR1* knockdown cells showed significantly less proton leak compared with controls; proton leak was decreased by 40.4% and 39.9%, respectively (Fig 5J).

## Discussion

We present the first large-scale investigation into gene regulators of body fat distribution in adipose tissue in a sex- and depot-specific manner. We constructed large Bayesian networks using male and female adipose tissue gene expression from subcutaneous and visceral samples, and we identified over 300 putative regulators spanning both sexes and depots. Using additional evidence, we prioritized 53 unstudied key driver genes that may affect adipocyte function and that putatively regulate body fat distribution. Because of the unbiased nature of our initial key driver selection, we were able to prioritize putative candidate GWAS genes, as well as putative causal genes that are not in GWAS loci.

In our study, we considered 495 genes nearby ∼400 WHR_adjBMI_ GWAS loci (Pulit et al, 2019) as candidate causal regulators of fat distribution. Other researchers have found that the nearest genes are likely, but not certainly, the mechanistic gene within the locus (Nasser et al, 2021), and problems identifying the causal gene at a locus often stem from multiple genes or signals (Franzén et al, 2016). Status as a replicated key driver provided independent, expression-based evidence that allowed us to prioritize 41 genes nearby WHR_adjBMI_ GWAS loci as putatively mechanistic, including *COL8A1*, *PSME3*, and *ANAPC2*. We showed that *PSME3* and *ANAPC2* have novel functional effects on adipogenesis; *COL8A1* was also prioritized in the colocalization studies by Raulerson et al (2019) and is likely a functional regulator of fat distribution as well. Of the 45 key driver genes already well characterized in adipocyte fat storage (Figs 2C and S8), 12 were nearby WHR_adjBMI_ GWAS loci. By integrating gene expression and WHR_adjBMI_ GWAS data, we can narrow the candidate causal gene list in an unbiased way.

Our network analyses were able to capture known biology, as well as make novel predictions. Most of the 45 well-studied key driver genes affected adipocyte fat storage by modulating adipogenesis, whereas a smaller number affected thermogenesis or lipolysis. Well-studied key driver genes *ACO1*, *ACAT1*, and *SLC25A1* are regulated in trans by KLF14; these genes may mediate KLF14’s effects on female fat distribution. Because of the bulk nature of our adipose tissue gene expression datasets, we identified key drivers in other cell types and pathways that were not fully explored here. *ARHGEF12* was identified as a key driver in all networks, making it an interesting candidate, although it was not prioritized here because it is not a candidate GWAS gene nor does it have strong effects in mice (Table S6).

Our analyses focused on 23 novel genes in two well-established pathways, Wnt signaling, and mitochondrial function, as putative drivers of adipocyte function; we demonstrated a functional role for five of the genes in these pathways. Two genes, *PSME3* and *RSPO1*, showed antagonistic effects on canonical and Ca^2+^ non-canonical Wnt signaling (Fig 4), consistent with previous studies (Akoumianakis et al, 2022). We showed that RSPO1 likely controls adipogenesis through its effects on Wnt signaling, consistent with the role of other r-spondins (Loh et al, 2020). RSPO1 is a novel serum marker of obesity (Kang et al, 2019) and may have a role in adipocyte beiging as well (Sun et al, 2023). However, Wnt signaling is unlikely the mechanism by which *PSME3* controls adipogenesis, because of the directionally inconsistent effects. PSME3 is a member of the 20S proteasome (Funderburk et al, 2022) known to degrade GSK3β (Li et al, 2015), consistent with our results that show lower levels of active:total GSK3β in *PSME3* overexpressing cells. Other 20S proteasome members affect fat storage (Kitamura et al, 2011; Willemsen et al, 2022), whereas *PSME3* itself has diverse roles in many cell types (Dong et al, 2013; Funderburk et al, 2022; Lee et al, 2023). Although the implications in adipocytes warrant further study, non-canonical Wnt ligands cause inflammation and vascular disease (Akoumianakis et al, 2022) and are released from visceral fat more than subcutaneous fat (Fuster et al, 2015). *ANAPC2* inhibited adipogenesis, although the loss of *ANAPC2* overexpression on day 12 or lack of Wnt signaling changes may account for the small change in adipogenesis observed. *ANAPC2* encodes part of the anaphase-promoting complex/cyclosome (APC/C) (Yu et al, 1998), which regulates the cell cycle, and others show that manipulating the expression of other APC/C proteins has consistent effects on adipogenesis (Lee et al, 2009; Du et al, 2022). Interestingly, we saw no differences in proliferation because of *ANAPC2* overexpression (Fig 3). MIGA1 may be involved in thermogenesis because of its broad effects on mitochondrial function (Fig 5). Four validated key driver genes, *MIGA1*, *ANAPC2*, *PSME3*, and *RSPO1*, have a role in fertility and the development of gonads or gametes (Tomizuka et al, 2008; McGuinness et al, 2009; Huang et al, 2016; Liu et al, 2016; Geng et al, 2020), indicating a potentially sex-specific role. Three validated key driver genes, *UBR1*, *ANAPC2*, and *PSME3*, have known roles in protein degradation (Yu et al, 1998; Tran, 2019; Funderburk et al, 2022), which may be another important pathway regulating adipocyte function.

Fat distribution is a complex, full-body phenotype that is difficult to recapitulate in vitro and in vivo models. We used subcutaneous and visceral gene expression data to make predictions, but we tested those predictions on the cells available to us, a subcutaneous male pre-adipocyte cell line and primary subcutaneous pre-adipocytes from a female donor. To fully characterize the effects of these genes on adipocyte function and fat distribution, we need to repeat experiments in both sexes and compare these results with visceral cells. We hypothesize that some will have larger effect sizes on visceral cells or female cells.

The regulation and presentation of fat distribution differ between males and females, and similarly, the in silico gene regulatory structures captured by the networks were sex-specific. Of the 53 prioritized key drivers, only five genes are replicated key drivers in both male and female networks. Although it was unsurprising to find that the predicted key drivers were different, we found that the male networks could predict almost twofold more key driver genes than the female networks. For male networks, this was significantly more key drivers than expected by random chance (Supplemental Results). We found that the difference in sample size between males and females could partially explain the difference in predictive power. Neither age and apparent menopausal status, nor expression of female regulator gene *KLF14* could explain the remaining differences in predictive power (Supplemental Results).

Our analysis compared two publicly available, independent datasets, STARNET and GTEx. There are large-scale differences between donors in these datasets, including living versus deceased donors, abdominal versus lower body subcutaneous adipose collection sites, cardiovascular disease status, age, ethnicity, BMI, and others (see the Materials and Methods section). These differences are reflected in the gene expression, which creates networks that are partially “overfit” to these samples, leading to the identification of key drivers that regulate dataset-specific processes. Accordingly, we find many key drivers shared between networks from the same dataset (Supplemental Results). By comparing the key drivers identified in STARNET and GTEx networks, we hope to filter out these dataset-specific key drivers, and identify genes that regulate the fat storage and homeostatic processes in the adipose tissue. Whereas our analysis successfully identified five genes with a role in adipocytes, some key drivers predicted by the networks were false positives and had no effect on adipogenesis or mitochondrial function.

We showed the validity and strength of Bayesian network modeling to predict known and novel gene regulators. We provided additional evidence of the role of Wnt signaling and mitochondria in adipocyte function and putatively in body fat distribution. Finally, we hypothesize a broader role for five genes in regulating fat distributions in humans.

## Materials and Methods

### Gene expression data

We interrogated RNA-sequencing gene expression data from subcutaneous adipose tissue and visceral abdominal adipose tissue from the GTEx (The Genotype-Tissue Expression, 2020) and the STARNET (Franzén et al, 2016). Detailed explanations of participant inclusion, data collection, sequencing, and quantification can be found at each source. Briefly, STARNET participants are people living with coronary artery disease, from whom biopsies of abdominal subcutaneous fat and abdominal visceral fat were obtained during open thorax surgery. Samples were sequenced using the Illumina HiSeq 2000 platform. GTEx biopsies of abdominal visceral fat and leg subcutaneous fat were taken from deceased donors shortly after death. Samples were sequenced using the Illumina TruSeq platform. Both datasets were obtained in transcripts per million format.

### Expression data processing

We first used annotation meta-data from each source to divide the data into males and females. We used *XIST* expression to confirm these assignments. Next, we used annotations from the R package bioMart for genome build hg38 to select only the protein coding genes within each dataset. We then removed genes with less than 0.1 transcripts per million value in greater than 80% of the samples. Finally, we log transformed the gene expression values for subsequent analysis.

### Bayesian network theory

Bayesian Networks are a type of Directed Acyclic Graph, where the relationships between the nodes in the graph are causal, or directed. Bayesian Networks have been used to determine directed connections between genes in an unbiased manner (Zhang et al, 2013, 2020; Mäkinen et al, 2014; Hamed et al, 2015; Huan et al, 2015; McKenzie et al, 2017; Shu et al, 2017, 2019; Agrahari et al, 2018; Chella Krishnan et al, 2018; Carcamo-Orive et al, 2020; Hartman et al, 2021). Based on Bayes’ theorem, the probability that a node in the network has a certain expression value depends on the expression of its parent nodes, or the nodes upstream of it. For gene A, with parent genes B and C, the probability that A has a particular expression value depends on the expression state of B and C:

$$P\left(A\right)=P\left(A|B,C\right)=P\left(A|B\right)•P\left(A|C\right)$$

Each edge in the network represents one of these conditional relationships between genes. The total structure of the graph describes all relationships between genes as a joint probability. The joint probability of a full graph, X, is described as the geometric sum of all individual node probabilities.

$$P\left(X\right)=\prod P\left(X_{i}|D_{i}\right)$$

where X is the full graph, X_i_ is a node in the graph X, and D_i_ is the set of parents for node X_i_.

Popular methods to learn the relationships within the graph generally try to maximize a likelihood function, which increases the ability of the graph’s joint probability to describe the observed gene expression data. Because multiple graph structures can result in the same likelihood, we also add prior information to improve predictions, such as known direct connections or eQTL data.

Bayesian network construction is a time and computationally intensive process, and few popular construction tools are able to handle networks with more than 100 nodes. The Reconstructing Integrative Molecular Bayesian Network (RIMBANET) (Zhu et al, 2004, 2008) tool is able to handle up to 10,000 nodes by discretizing the gene expression data to reduce computational complexity. RIMBANET also takes in diverse prior information, including direct connections, eQTL data, and continuous gene expression data, to improve the predictive power of the network. We chose RIMBANET to construct Bayesian networks for its ability to handle large input genesets and its reproducibility between datasets (Zhu et al, 2007; Cohain et al, 2017).

### Bayesian network input genes

Ideally, we would probe gene-gene interactions at a genome-wide level, but Bayesian network construction is computationally intensive, and therefore, we limited this analysis to a subset of <10,000 genes that are more likely to regulate body fat distribution (Fig 1). We prioritized putative regulators of body fat distribution using three strategies: (1) genes whose expression are co-expressed with others in adipose tissue, (2) genes proximal to body fat distribution GWAS loci (Pulit et al, 2019), and (3) genes that are putatively regulated by the transcription factor KLF14 (Small et al, 2018).

#### Co-expressed genes

For a gene to be connected to others in a co-expression network, it must be expressed in the measured dataset, must vary between samples, and must be correlated with the expression of other genes. These properties are optimal for Bayesian network construction and can indicate gene function in the tissue of interest; therefore, we constructed adipose tissue co-expression networks for all eight datasets and identified genes connected to the corresponding STARNET and GTEx networks. We used the python package iterativeWGCNA (Greenfest-Allen et al, 2017 *Preprint*) to obtain modules of co-expressed genes in each dataset. Weighted gene co-expression network analysis (Langfelder & Horvath, 2008) uses correlations found within the data to determine which groups of genes are highly correlated and likely co-regulated. First, we computed the correlations between all genes. We raised these correlation coefficients to an empirically determined power to increase the differences observed. Next, we performed hierarchical clustering on the correlation matrix to define modules of highly correlated genes. We then assessed the success of this clustering, and iteratively reassigned genes to the modules in which they fit best. Lowly expressed or uncorrelated genes were not assigned a module. We identified which genes were assigned to modules in each of the eight datasets. We then compared the GTEx and STARNET module assignments for each depot and sex; we found genes assigned to modules in both datasets in the four depot and sex groups. We then took the union set of these four genesets as the co-expressed geneset, contained 7,928 genes and made up the bulk of the input to Bayesian network construction (Table S1).

#### *KLF14* trans-*eQTL* network genes

We have previously demonstrated that *KLF14* expression regulates fat distribution in both female mice and humans (Yang et al, 2022). SNP rs4731702 is significantly associated with KLF14 expression in cis in adipose tissue of multiple cohorts (Civelek et al, 2017; Small et al, 2018). The same variant is also associated with the expression of 385 genes across the genome in trans- (Table S1). We hypothesized that *KLF14*’s effect on fat distribution is mediated by the genes it regulates, and we included 385 KLF14 putative target genes in the input geneset.

#### *WHR*_*adjBMI*_ *GWAS* loci-adjacent genes

The largest WHR_adjBMI_ GWAS meta-analysis to date was performed on primarily European ancestry and discovered 346 loci associated with WHR_adjBMI_ (Pulit et al, 2019). Multiple sources have determined that the functional gene is the nearest gene to the locus in ∼70% of cases (Nasser et al, 2021), so we identified 443 genes overlapping or nearest to the lead SNP (and SNPs with LD r^2^ > 0.8) of 346 WHR_adjBMI_ GWAS loci using haploReg (Ward & Kellis, 2016). Further, we used two studies that identified high-quality candidate genes using colocalization methods (Civelek et al, 2017; Raulerson et al, 2019), where the SNPs that affect association with WHR_adjBMI_ also affect the expression of 59 candidate genes, which are more likely to be functional (Table S1). In total, we considered this combined set of 495 genes as WHR_adjBMI_ GWAS genes in this study. Whereas this set does not contain all possible causal genes, it is likely enriched for them.

The union set of weighted gene co-expression network analysis module genes, KLF14 targets, and putative GWAS genes made up the input to Bayesian Network construction. For each dataset, the 8,492 gene expression values were discretized into “low” “medium” and “high” bins using k-means clustering.

### Prior information

Because multiple graph structures can result in the same likelihood score, we can use prior information to improve confidence in the network structure.

#### eQTLs

After the central dogma, information flows from DNA to RNA; therefore, if SNPs influence the expression of gene A, gene A is more likely to be regulatory of other genes B and C, etc. For each dataset, we determined which genes had *cis*-eQTLs with SNPs < ±500 kb. These eGenes were more likely to be parent nodes in the Bayesian networks. Neither STARNET nor GTEx determined *cis*-eQTLs in a sex-specific manner, so these eQTL eGenes were nearly identical for male and female networks (Table S2).

#### Continuous data

Although the network is built on discretized gene expression data, RIMBANET is able to use continuous gene expression data to inform network construction. First, the continuous data are used to generate Pearson correlations between all genes. Correlations with significance *P* < 0.01 are used as prior information to determine possible parents and prioritize which edges to add or remove.

### Bayesian network construction

Bayesian Networks for each dataset were constructed using RIMBANET using the discretized gene expression data, a list of eQTL eGenes, and the continuous gene expression data. The RIMBANET shell script was adapted for implementation on the University of Virginia’s high-performance computational cluster (Rivanna). RIMBANET was run with these tags: –C TRUE to specify continuous data, -w to add the continuous dataset, -d to add the discretized dataset, -e to add eQTL eGenes. RIMBANET creates 1,000 versions of each network with random initial seeds. RIMBANET uses a Markov blanket to identify potential parents for each gene, then iteratively adds edges with the highest prior information to create the best network structure to represent the data, as measured by the Bayesian Information Criterion (BIC).

BIC=k•ln(n)−2•ln (L)

where n is the number of nodes, k is the number of parameters estimated (edges), and L is the likelihood function. Maximizing this function results in the best fit graph structure that is not overly complex. Then, RIMBANET merges the 1,000 network versions by retaining edges present in 30% of the iterations. Finally, RIMBANET produces a directed acyclic graph by removing complete cycles.

### Properties of biological networks

Biological networks share some common features, including reproducibility, scale-freeness, and small-worldness (Takahashi et al, 2012). Scale-free networks have degree distributions that follow the power law; most nodes have a small number of downstream genes, whereas a few nodes have a large number of downstream genes (Barabasi & Albert, 1999). The distribution of the number of downstream genes per parent can be fitted as a power law P(k) ∼ k^−a^, where k represents the number of downstream edges and P(k) represents the fraction of nodes that have k edges. The degree exponent, a, is determined by fitting the line log(P(k) ∼ −a log(k). In biological networks, the degree exponent is commonly 2–3. We used the igraph() package in R to calculate the degree exponent for each network. Small-world networks are highly clustered yet have a short average distance between nodes (Watts & Strogatz, 1998). We used the qgraph() package in R to calculate the clustering coefficient and average path length between nodes for each network. Because these properties scale with the number of nodes in the network, we compared these metrics with a random graph of the same size.

### Key driver gene analysis

Key driver genes are genes that, because of their prominent position in the network, regulate many genes downstream. We were particularly interested in identifying key driver genes that regulate disease-related genes, specifically WHRadjBMI GWAS genes. We hypothesize that these in silico key driver genes also regulate disease-related gene programs in vitro.

Key driver genes of each network were identified with two methods: (1) by the number of downstream genes regulated by each potential key driver gene and (2) by the enrichment of disease genes in the set of downstream genes regulated by each potential key driver gene.

To identify type 1 key driver gene testing, first, every gene in the network was profiled to determine its number of downstream genes at distances 1–10 edges away using the shortest path. Then, the mean and SD in the number of downstream genes at each edge distance were calculated for the network.

For each potential key driver gene, we calculated 10 score functions:

$${Score}_{dist=n}=\left(G_{dist=n}-{NM}_{dist=n}\right)/{NS}_{dist=n}$$

where n is the distance in edges, G is the number of downstream genes the potential key driver gene has at distance n, NM is the network mean number of downstream genes at distance n, NS is the network SD in the number of downstream genes at distance n. This is a metric of the extremeness of G, effectively a z-score.

Finally, we calculated a total score for each potential key driver gene by summing the “z-scores,” weighting smaller edge distances away from the potential key driver higher than large edge distances:

$$GeneScore={Score}_{dist=1}•1+{Score}_{dist=2}•1+{Score}_{dist=3}•1+{Score}_{dist=4}•0.75+{Score}_{dist=5}•0.5+{Score}_{dist=6}•0.25+{Score}_{dist=7}•0.125+{Score}_{dist=8}•0.0625+{Score}_{dist=9}•0.03125$$

The top 10% the highest scoring genes in the network were declared type 1 key driver genes, similar to previous studies (Zhang et al, 2013).

To identify type 2 key driver genes, a “neighborhood” of downstream genes was declared for every gene in the network as those genes within four edges away. Next, WHR_adjBMI_ GWAS genes (Pulit et al, 2019), defined above, were identified within the downstream neighborhood. Finally, using Fisher’s exact test, we determined whether the number of GWAS genes in the downstream neighborhood was significantly more than expected by chance, compared with the whole network. Genes with significant downstream enrichments of WHR_adjBMI_ GWAS genes were declared type 2 key driver genes.

Key driver genes for each network were the union set of type 1 and type 2 key driver genes. Shared key driver genes were genes identified as either type 1 or type 2 key driver genes in both STARNET and GTEx networks of the same type.

### Testable key driver gene selection

Whereas this set of 334 replicated key driver genes likely contains many novel mechanistic drivers of fat distribution or fat storage, it also likely contains false positives and well-characterized genes. Furthermore, we hypothesize that fat distribution is driven, in part, by adipocyte expansion, and while genes involved in other adipose tissue processes, such as tissue structure, immune function, vascularization, etc., might contribute to fat distribution or its comorbidities, these were not the focus of this study. We used three steps to narrow this list to likely functional, testable adipocyte key driver genes.

#### Identification of cell type

We used seven publicly available single cell- or single nucleus-, adipose tissue- or adipose tissue-derived stromal vascular fraction RNA-sequencing datasets from both human and mouse (Hepler et al, 2018; Min et al, 2019; Almanzar et al, 2020; Sun et al, 2020; Vijay et al, 2020; Hildreth et al, 2021; Sárvári et al, 2021) to determine the primary cell type in which the gene was expressed. Because there was some disagreement between studies, all cell types in which the gene was expressed in any study are reported in Table S6. We removed genes that were only expressed in non-adipocyte cell types.

#### Identification of function in adipocytes

We used a comprehensive literature search to identify well-studied key driver genes (Supplemental Data 1). For each gene, we used GeneCards to identify alternate names for each gene or corresponding protein. We then searched PubMed and Google Scholar for functional studies in cells demonstrating a role for that gene in pre-adipocytes or adipocytes. Terms searched include “adipocyte,” “adipogenesis,” “differentiation,” “lipogenesis,” “lipolysis,” “glucose uptake,” “browning,” and “thermogenesis.”

#### Additional genetic evidence

We prioritized key driver genes based on two types of genetic evidence. First, we identified 41 WHR_adjBMI_ GWAS genes (Pulit et al, 2019) within the set of remaining key driver genes. Their identity as a regulator of genes within the network and their location near a significant GWAS locus is strong evidence that they likely have a functional role in fat distribution. Second, we prioritized genes involved in fat storage and distribution in mouse models. We queried the Mouse Genome Informatics database (Blake et al, 2021) and the International Mouse Phenotyping Consortium (Groza et al, 2023) to determine if the gene knockout in mice results in significant differences in fat pad size, total body fat mass, lean mass, or related phenotypes. We did not consider overall body size differences, as these may be indicative of BMI-related phenotypes. Although there are differences in fat storage between mice and humans, there are many conserved pathways that point to shared genetic mechanisms and similar biological outcomes (Chusyd et al, 2016; Börgeson et al, 2022). We hypothesized that they play a similar role in human fat storage.

### Lentivirus construction

Overexpression plasmids were constructed by VectorBuilder (VectorBuilder Inc) using the mammalian gene expression lentiviral vector backbone with one open reading frame. This backbone contains third generation lentiviral integration sites and ampicillin resistance. Using GTEx, we identified the most abundant isoform in adipose tissue for each gene. This isoform was added to the plasmid, followed by a P2A linker, then the GFP reporter sequence. This construct was under the CMV promoter. Control plasmids contain the GFP reporter gene under the CMV promoter with no P2A linker.

7TFC was a gift from Roel Nusse, purchased from Addgene (Addgene plasmid # 24307; Addgene). The 7TFC plasmid contains third generation lentivirus integration sites, the Firefly Luciferase gene under seven repeats of the TCF promoter, an mCherry marker under the SV40 promoter, and the ampicillin bacterial resistance gene.

We obtained the plasmids in *E. coli* swabs in agar. We cultured the *E. coli* on Luria-Bertani broth agar plates containing 100 μg/ml ampicillin and sub-cultured single colony-forming units in 50 ml Luria-Bertani broth containing 100 μg/ml ampicillin. Plasmids were isolated from *E. coli* using the Nucleobond Xtra Midi prep kit (Takara Bio) following the manufacturer’s protocol (Cat# 740422.50). Plasmids were packaged into third generation replication-deficient lentivirus in HEK-293T cells using the Lenti-Pac HIV Expression Packaging Kit (Genecopoeia) following manufacturer’s protocol (Cat# LT001).

### Transduction and sorting of human pre-adipocyte overexpression cell lines

We obtained human male pre-adipocyte Simpson-Golabi-Behmel syndrome (SGBS) cells from Dr. Martin Wabitsch (Wabitsch et al, 2001) at passage number 35. There are no cell lines for female or visceral pre-adipocytes; only models using primary cells are available (Ruiz-Ojeda et al, 2016). All pre-adipocyte cells were grown and maintained as described previously (Fischer-Posovszky et al, 2008) in DMEM:F12 media (Thermo Fisher Scientific) containing 10% FBS, 1% Penicillin/Streptomycin, 8.1 ng/ml biotin and 3.5 ng/ml pantothenate. During transduction with lentivirus, the FBS was first heat-inactivated at 65°C for 30 min, and 8 μg/ml polybrene was added to improve transduction efficiency. We plated cells in 6-well plates and grew them to 70% confluence before transducing the cells with lentiviral particles containing each of the plasmids listed above. Cells containing high levels of GFP or mCherry were sorted using the FACS Aria Fusion Cell Sorter (BD Biosciences). We used cells with a passage number less than 46 for subsequent assays.

### Transfection and differentiation of human primary pre-adipocytes

Human female subcutaneous primary pre-adipocytes were purchased from Zenbio (Cat# SP-F-SL; Lot# SL0061; Zenbio) and were differentiated according to the standard Zenbio white adipocyte differentiation protocol. Briefly, human primary subcutaneous pre-adipocytes were seeded on collagen-coated 96-well plate (20,000cell/well, 354650; Corning) with 200 μl of PM-1 medium (#PM-1; Zenbio), and the cells were established overnight, then transfected with siRNAs of target genes (Table 5) or scramble controls for 3 d using Lipofectamine RNAiMAX (cat# 13778-150; Invitrogen). The culture medium was replaced with 150 μl of differentiation medium DM-2 (#DM-2; Zenbio), and cells were cultured for 7 d. Media was replaced with maintenance medium AM-1, and cells were cultured for an additional 7 d for the following qRT-PCR and Seahorse experiments.

**Table 5.** siRNA sequences.

Gene symbol	Sequence
A4GALT	GGACACGGACUUCAUUGUU
A4GALT	GCACUCAUGUGGAAGUUCG
A4GALT	AGAAAGGGCAGCUCUAUAA
A4GALT	UGAAAGGGCUUCCGGGUGG
BAD	GAUCGGAACUUGGGCAGGG
BAD	CAGAGUUUGAGCCGAGUGA
BAD	GAGCUCCGGAGGAUGAGUG
BAD	UUGUGGACUCCUUUAAGAA
C1QTNF3	CCGCAAAUUCUAAAUCUUA
C1QTNF3	UCAACCUAGUAGAGGACAA
C1QTNF3	AGGUGAGAAGGGCGACAAA
C1QTNF3	CAGUAUCAGGUGUGUAUUU
MIGA1	GCUCUGACCUUUCGCAAUA
MIGA1	ACACAGAGAAGUACGGCAU
MIGA1	GGAAAUAUCUCUUUAUCGU
MIGA1	CUUGAAGACAGCAGCGCUA
PSME3	GAAUCAAUAUGUCACUCUA
PSME3	UCUGAAGGAACCAAUCUUA
PSME3	GCUAAGAACUGUUGAGAGU
PSME3	GACCAGAUUUCUAGAUAUU
UBR1	GGAAAUCAGCGCGGAGUUA
UBR1	GUACAAUCGUGUGGACAUA
UBR1	GCGAAGAAAUGGACUGUCU
UBR1	GAUCAGCAAACCCACAAUA
ZNF148	UGGAAUAGCUACUCAAUUU
ZNF148	UUGAAUAGCCCGAGCCUUA
ZNF148	GGAUCAAGCUCCCAAGCAU
ZNF148	CUAAGAACAACUCCAGAUA
TRIP12	GAACACAGAUGGUGCGAUA
TRIP12	GACAAAGACUCAUACAAUA
TRIP12	GCUCAUAUCGCAAAGGUUA
TRIP12	GGUAGUGACUCCACCCAUU

### Quantification of gene expression in lentiviral treated human pre-adipocytes and differentiating cells

We grew cells in 12-well plates. Once they reached confluency, they were washed with PBS and incubated in 400 μl Trizol, then scraped and harvested. We extracted RNA using the RNeasy Micro Kit (QIAGEN), following the manufacturer’s protocol (Cat# 74004). We digested the DNA species on the QIAGEN spin column using the RNAse-free DNAse kit (QIAGEN) following manufacturer’s protocol (Cat# 79254). We quantified the isolated RNA using the Qubit with an RNA Broad Range assay kit (Thermo Fisher Scientific), following the manufacturer’s protocol (Cat# Q10210). We reverse transcribed cDNA from the RNA templates using SuperScript IV Reverse Transcriptase Kit (Thermo Fisher Scientific) with Oligo(dT)20 primers, following the manufacturer’s protocol (Cat# 18090010). We quantified cDNA abundance using qRT-PCR. Samples and standard curves were prepared using GoTaq qRT-PCR Master Mix (Promega) and gene specific primers (Integrated DNA Technologies) (Table 6). Samples were measured using the QuantStudio 5 Real-Time PCR system (Thermo Fisher Scientific), and were analyzed using Thermo Fisher Scientific Connect qRT-PCR Standard Curve analysis software.

**Table 6.** qRT-PCR primer sequences.

Primer name	Sequence
Primers used in adipogenesis/Wnt signaling assays	
ADIPOQ F	GGAGATCCAGGTCTTATTGGTCC
ADIPOQ R	GCACCTTCTCCAGGTTCTCC
ANAPC2 F	GCGAGAAGAAGTCCACACTATG
ANAPC2 R	GACTCTCAAGAAGCACCCATAC
AXIN2 F	GACCAAGTCCTTACACTCCTTATT
AXIN2 R	TCTAAGGTATCCACGCATTTCTC
B2M F	AGATGAGTATGCCTGCCGTGT
B2M R	TGCTGCTTACATGTCTCGATC
CEBPA F	TATAGGCTGGGCTTCCCCTT
CEBPA R	AGCTTTCTGGTGTGACTCGG
IL6 F	CCAGGAGAAGATTCCAAAGATGTA
IL6 R	CGTCGAGGATGTACCGAATTT
PPARG F	ACCCAGAAAGCGATTCCTTCA
PPARG R	TCCACTTTGATTGCACTTTGGT
PSME3 F	CTGAGATCCGGCTGTTGATT
PSME3 R	CAGGAGCTGTACCCACATTT
RSPO1 F	GTGAAATGAGCGAGTGGTCT
RSPO1 R	GTAGCACCCTGCGTGTC
RSPO3 F	GAAACACGGGTCCGAGAAATA
RSPO3 R	CCCTTCTGACACTTCTTCCTTT
TYRO3 F	GAGTGTATGGAGGACGTGTATG
TYRO3 R	GTTCCATTCGCAGACAAGTAAAG
Primers used in mitochondrial function assays	
A4GALT-F	GCATCTACCTGGACACGGACTT
A4GALT-R	ATGCACAGCGCCATGAACTCGT
AKAP9-F	GGCGTCATTGATGGCTATGCAG
AKAP9-R	GCTGTTGCTCTGCCTCCAATTC
ARAP1-F	GTGTGGACTACATCACGCAGTG
ARAP1-R	CCGAGGAAACATCATCCACGTG
BAD-F	CCAACCTCTGGGCAGCACAGC
BAD-R	TTTGCCGCATCTGCGTTGCTGT
C1QTNF3-F	GGAGACTACAGCTTTCGAGGCT
C1QTNF3-R	TTGTCGCCCTTCTCACCTTTGG
LDHD-F	GATGGATGCCTGCAACAGGTAC
LDHD-R	TCCGTTCTGCTGGACTATCTCC
MIGA1-F	GTGCAGGAGATGCCATTGCTGA
MIGA1-R	ACTCCTCTTGGAGACGATAGGC
PSME3-F	ATGAATCTCCCAGTCCCTGACC
PSME3-R	GGGCATCACAAACACCTTGGTTC
QTRT1-F	GTAGTCTGCGTGGCTCTTGGAT
QTRT1-R	GCCGAAGTCCTTCTCAAACACC
UBR1-F	GTAGCAACCACATCAGGATCGG
UBR1-R	CTGTAAGGCAGACATCTGAGCC
ZNF148-F	CACGTTTGTGAGCACTGCAATGC
ZNF148-R	GCAGGTACTTCTGTATGAAACGC
TRIP12-F	GGCTGCCTCAAAGGATACCATC
TRIP12-R	GCAGCACAAAGTCTCTGAAGGAC
GAPDH-F	GTCTCCTCTGACTTCAACAGCG
GAPDH-R	ACCACCCTGTTGCTGTAGCCAA
hCEPBA-F	ACAAGAACAGCAACGAGTACCG
hCEPBA-R	CATTGTCACTGGTCAGCTCCA
hFABP4-F	ACGAGAGGATGATAAACTGGTGG
hFABP4-R	GCGAACTTCAGTCCAGGTCAAC
hUCP1-F	AGTTCCTCACCGCAGGGAAAGA
hUCP1-R	GTAGCGAGGTTTGATTCCGTGG
hPPARG-F	AGCCTGCGAAAGCCTTTTGGTG
hPPARG-R	GGCTTCACATTCAGCAAACCTGG

### Quantification of gene expression in siRNA-treated human differentiated adipocytes

The total RNA samples were isolated from human differentiated adipocytes using KingFisher Flex Magnetic Particle Processor according to the MagMAX mirVana Total RNA isolation protocol. We obtained the cDNA samples from the RNA templates using SuperScript IV Reverse Transcriptase Kit (Thermo Fisher Scientific) with Oligo(dT)20 primers, following the manufacturer’s protocol (Cat# 18090010). We quantified cDNA abundance using qRT-PCR using the QuantStudio 5 Real-Time PCR system (Thermo Fisher Scientific). The conditions were: 42°C for 5 min, a 10 s denaturation step at 95°C, followed by 40 cycles of 95°C for 5 s and 58°C for 40 s.

### Cellular phenotyping

#### Proliferation assay

We plated 20,000 cells/well in 24-well plates. After 24 h, four wells were trypsinized, and cells were counted using a hemocytometer. Wells were washed with PBS and growth media was replaced. Every 24 h, four more wells were counted, for a total of 6 d. Growth media was replaced every 2 d. Most cells reached exponential growth by day 4 (Fig 4B). We then calculated the doubling time of each cell line using the formula:

$$T_{d}=\left(T_{2}-\;T_{1}\right)•\left[\frac{\ln\left(2\right)}{\ln\left(\frac{N_{2}}{N_{1}}\right)}\right]$$

where T_d_ is doubling time, T_1_ and T_2_ are initial and final time measurements, and N_1_ and N_2_ are the initial and final quantity of cells.

#### Adipogenesis assay

We differentiated cells into lipid-containing adipocytes as detailed previously (Fischer-Posovszky et al, 2008). Briefly, we plated 40,000 cells/well in 12-well plates. Cells were incubated for 2–5 d until they reached 100% confluency, then incubated for 48 h post-confluency. Adipogenic media (DMEM:F12, 1% Penicillin/Streptomycin, 8.1 ng/ml biotin, 3.5 ng/ml pantothenate, 0.01 mg/ml transferrin, 20 nM insulin, 100 nM cortisol, 0.2 nM triiodothyronine, 25 nM dexamethasone, 250 μM 3-isobutyl-1-methylxanthine, and 2 μM rosiglitazone) was added to each well to initiate differentiation. After 4 d, we changed the media to DMEM:F12, 1% Penicillin/Streptomycin, 8.1 ng/ml biotin, 3.5 ng/ml pantothenate, 0.01 mg/ml transferrin, 20 nM insulin, 100 nM cortisol, and 0.2 nM triiodothyronine. Every 4 d, this media was replaced.

#### Quantification of adipogenesis

We quantified the amount of lipid stored in cells using Oil Red O (ORO) dye. 0.25*g* of dye was suspended in 48 ml of 98% isopropanol and 32 ml of DI water. Unsuspended dye was removed from the ORO solution using 0.045 μM vacuum filtration. We repeated filtration (∼3X) every 24 h until no precipitate was observed. Cells were washed with PBS, then fixed at 300 μl 4% PFA for 15 min. Cells were washed with 60% isopropanol, then dried completely. 250 μl of ORO solution was added to the cells for 5 min. Cells were washed with DI water twice, then dried completely. We imaged the full wells using the EVOS microscope (below). ORO dye was then eluted from cells in 200 μl of 100% isopropanol for 2 min. Eluted ORO was quantified by measuring absorbance at 450 nm.

#### Imaging

We took images using the EVOS M7000 imaging system (Thermo Fisher Scientific) at 10x magnification, using phase contrast and color. We constructed full well composite images by taking 30 adjacent images in a 5 × 6 grid that covers most of the well. Composite images were stitched together using imageJ:Fiji plugin Grid/Collection Stitching (Schindelin et al, 2012).

### Quantification of Wnt signaling

#### Quantification of Wnt signaling transcriptional activation

We performed luciferase assays using the SGBS:7TFC reporter line. SGBS:7TFC cells were transduced with lenti-virus containing the gene of interest or GFP control plasmids. Images were taken to ensure a high percentage of dual mCherry and GFP-expressing cells. 10,000 cells/well were plated in clear bottom, white-walled 96-well plates, with six replicates of each gene or control. After 24 h of incubation, luciferase activity was measured using the Luciferase Assay System (Promega) following manufacturer’s protocol (Cat# E1500). Briefly, the cells were lysed in a 20 μl lysis buffer, 100 μl of luciferin-containing reagent was added, then emitted light was measured for 10 s using a luminescence plate reader. Luminescence readouts in each well were normalized to mCherry fluorescence to account for total luciferase insertions by the 7TFC cassette.

#### Quantification of protein activation

Active (Ser33/Ser37/Thr41 non-phosphorylated) β-catenin and total β-catenin species were measured using Western blotting. Total proteins were isolated in RIPA buffer containing 1% protease and 1% phosphatase inhibitors (Cat# 89901, Cat# 78429, Cat# 78426; Thermo Fisher Scientific). We quantified total protein species using the bicinchoninic acid (BCA) assay (Thermo Fisher Scientific) following the manufacturer’s protocol (Cat# 23225). We denatured samples at 70°C for 10 min, then ran 20 μg total protein on a NuPAGE 4–10% BisTris Gel at 240 V for 40 min (Cat# NP0336BOX; Thermo Fisher Scientific). We transferred the protein to an Immobilon-FL PVDF membrane at 80 V for 60 min (Cat# IPFL00010; MilliporeSigma). We labeled active and total β-catenin and β-actin control bands using primary antibodies (Non-phospho [Active] β-Catenin [Ser33/37/Thr41] [D13A1] Rabbit mAb Cat#8814, dilution 1:500; β-Catenin [15B8] Mouse mAb Cat#37447, dilution 1:1,000; Cell Signaling Technologies). Bands were labeled with fluorescently conjugated secondary antibodies (Goat anti-Mouse IgG [H+L] Cross-Adsorbed Secondary Antibody, Cyanine3, Cat# A10521, dilution 1:20,000; Goat anti-Rabbit IgG [H+L] Cross-Adsorbed Secondary Antibody, Cyanine3, Cat# A10520, dilution 1:20,000; Thermo Fisher Scientific). We imaged the labeled protein on Amersham Imager 600 (Global Life Sciences Solutions) using RGB fluorescence settings. Densitometry calculations were performed using imageJ.

The amount of active and total GSK3β, JNK, and CAMK2A were quantified using ELISA. Cells were harvested and lysed according to each manufacturer’s protocol. Active GSK3β (Ser9 phosphorylated) and total GSK3β were measured using an ELISA kit (RayBiotech) using manufacturer’s protocols (Cat# PEL-GSK3b-S9-T). Active JNK (Thr183/Tyr185 phosphorylated) and total JNK were measured using an ELISA kit (RayBiotech) using manufacturer’s protocols (Cat# PEL-JNK-T183-T-1). Active CAMK2A (Thr286 phosphorylated) and total CAMK2A were measured using an ELISA kit (Assay BioTechnology) using manufacturer’s protocols (Cat# FLUO-CBP1509 and CB5092).

### Quantification of OCR and ECAR

OCR and ECAR was determined using a Seahorse XF96 analyzer in combination with the Seahorse mitochondrial stress test kit according to a standard protocol (Ding et al, 2018). In brief, human primary pre-adipocytes were plated and differentiated as described above. Differentiated cells were washed with DPBS twice and incubated with Seahorse XF assay medium supplemented with 2 mM glutamax, 10 mM glucose, 1 mM sodium pyruvate (PH 7.4) for 45 min at 37°C in a non-CO2 environment. OCR and ECAR were subsequently measured in real time using XF96 extracellular flux analyzer (Seahorse Bioscience). The optimized concentration of compounds for mito-stress assay were 1.5 μM of oligomycin, 1.5 μM of carbonyl cyanide-p-trifluoromethoxyphenylhydrazone (FCCP), and 0.5 μM of Rotenone and antimycin A. After the extracellular flux analysis, the OCR and ECAR were normalized by cell number, quantified using Hoechst staining.

#### Quantification of deep OCR phenotypes

We calculated basal mitochondrial respiration, ATP-linked respiration, proton leak, maximal respiratory capacity, reserve capacity, and non-mitochondrial respiration from the OCR assay as described previously (Rose et al, 2014; Son et al, 2017). We defined condition “A” as timepoints 1, 2, and 3 under basal stimulation; condition “B” as timepoints 4, 5, 6 under oligomycin stimulation; condition “C” as timepoints 7, 8, 9 under FCCP stimulation; and condition “D” as timepoints 10, 11, and 12 under Rot/AA stimulation. We considered each of the three timepoints within each condition as technical replicates, and the six samples as biological replicates. We averaged the three timepoints per condition into one value per biological replicate. We then defined non-mitochondrial respiration as D; basal mitochondrial respiration as A–D; ATP-linked respiration as A–B, proton leak as B–D; maximal respiratory capacity as C–D; and reserve capacity as C-A.

### Statistical methods

Differences in proliferation and differentiation assays using the GFP-expressing control cells and cells expressing the gene of interest were assessed using two-way ANOVA by gene and time (day). Post hoc tests were performed between GFP controls and genes of interest within each timepoint using pooled *t* tests with *P*-value adjustment using Dunnett’s adjustment. Differences in Wnt signaling using the GFP-expressing control cells and cells expressing the gene of interest and mitochondrial assays using the non-targeting control-expressing cells and cells with siRNA for the gene of interest were assessed using one-way ANOVA by gene. Post hoc tests were performed between controls and genes of interest using pooled *t* tests with *P*-value adjustment using Dunnett’s adjustment. Analyses were performed using base R’s anova() function and the emmeans package’s emmeans() and contrasts(). We reported only significant *P*-values, with the exception of Fig 5 where indicated. All bar plots display the mean, with error bars displaying the SEM.

## Supplementary Material

Reviewer comments

## Data Availability

Gene expression and eQTL data from GTEx can be found at dbGaP Accession phs000424.v8.p2, accessed on 10/01/2020. Gene expression and eQTL data STARNET can be found at https://www.ncbi.nlm.nih.gov/projects/gap/cgi-bin/study.cgi?study_id=phs001203.v3.p1, data available on request. Code used in these analyses can be found at https://github.com/jnr3hh/Reed_Civelek_2023_manuscript.
