# Supplementary material for: Systems genetics analysis of human body fat distribution genes identifies adipocyte processes
Source: Life Sci Alliance. 2024 May 3;7(7):e202402603. doi: 10.26508/lsa.202402603 (PMC11068934; doi:10.26508/lsa.202402603)
Supplement: Supplementary file 8 [file LSA-2024-02603_Supplemental_Data_1.docx]

**Extended Bibliography 1- Literature Search 45 Adipocyte Genes:**

1. Ibrahim S, Temtem T. Medium-Chain Acyl-COA Dehydrogenase Deficiency. 2021 Jul 26. In: StatPearls
2. Lim SC, et al. Loss of the Mitochondrial Fatty Acid β-Oxidation Protein Medium-Chain Acyl-Coenzyme A Dehydrogenase Disrupts Oxidative Phosphorylation Protein Complex Stability and Function. Sci Rep. 2018 Jan 9;8(1):153.
3. Houten SM, Wanders RJ. A general introduction to the biochemistry of mitochondrial fatty acid β-oxidation. J Inherit Metab Dis. 2010 Oct;33(5):469-77.
4. Vega RB, Kelly DP. A role for estrogen-related receptor alpha in the control of mitochondrial fatty acid beta-oxidation during brown adipocyte differentiation. J Biol Chem. 1997 Dec 12;272(50):31693-9.
5. Huang LH, et al. Myeloid-specific Acat1 ablation attenuates inflammatory responses in macrophages, improves insulin sensitivity, and suppresses diet-induced obesity. Am J Physiol Endocrinol Metab. 2018 Sep 1;315(3):E340-E356.
6. Xu Y, et al. Enhanced acyl-CoA:cholesterol acyltransferase activity increases cholesterol levels on the lipid droplet surface and impairs adipocyte function. J Biol Chem. 2019 Dec 13;294(50):19306-19321.
7. Zhu Y, et al. In vitro exploration of ACAT contributions to lipid droplet formation during adipogenesis. J Lipid Res. 2018 May;59(5):820-829.
8. Moreno M, et al. Cytosolic aconitase activity sustains adipogenic capacity of adipose tissue connecting iron metabolism and adipogenesis. FASEB J. 2015 Apr;29(4):1529-39.
9. Yogosawa S, et al. Activin receptor-like kinase 7 suppresses lipolysis to accumulate fat in obesity through downregulation of peroxisome proliferator-activated receptor γ and C/EBPα. Diabetes. 2013 Jan;62(1):115-23.
10. Suchý T, et al. The repertoire of Adhesion G protein-coupled receptors in adipocytes and their functional relevance. Int J Obes (Lond). 2020 Oct;44(10):2124-2136.
11. Paik J, Haenisch M, Muller CH, Goldstein AS, Arnold S, Isoherranen N, Brabb T, Treuting PM, Amory JK. Inhibition of retinoic acid biosynthesis by the bisdichloroacetyldiamine WIN 18,446 markedly suppresses spermatogenesis and alters retinoid metabolism in mice. J Biol Chem. 2014 May 23;289(21):15104-17.
12. Kang S. Adipose Tissue Malfunction Drives Metabolic Dysfunction in Alström Syndrome. Diabetes. 2021 Feb;70(2):323-325.
13. Geberhiwot T, et al. Relative Adipose Tissue Failure in Alström Syndrome Drives Obesity-Induced Insulin Resistance. Diabetes. 2021 Feb;70(2):364-376.
14. Hearn T. ALMS1 and Alström syndrome: a recessive form of metabolic, neurosensory and cardiac deficits. J Mol Med (Berl). 2019 Jan;97(1):1-17.
15. Huang-Doran I, Semple RK. Knockdown of the Alström syndrome-associated gene Alms1 in 3T3-L1 preadipocytes impairs adipogenesis but has no effect on cell-autonomous insulin action. Int J Obes (Lond). 2010 Oct;34(10):1554-8.
16. Favaretto F, et al. GLUT4 defects in adipose tissue are early signs of metabolic alterations in Alms1GT/GT, a mouse model for obesity and insulin resistance. PLoS One. 2014 Oct 9;9(10):e109540.
17. Claussnitzer M, et al. FTO Obesity Variant Circuitry and Adipocyte Browning in Humans. N Engl J Med. 2015 Sep 3;373(10):895-907.
18. Lee KY, et al. The differential role of Hif1β/Arnt and the hypoxic response in adipose function, fibrosis, and inflammation. Cell Metab. 2011 Oct 5;14(4):491-503.
19. Kälin S, et al. A Stat6/Pten Axis Links Regulatory T Cells with Adipose Tissue Function. Cell Metab. 2017 Sep 5;26(3):475-492.e7.
20. Valerio CM, et al. Dipeptidyl peptidase-4 levels are increased and partially related to body fat distribution in patients with familial partial lipodystrophy type 2. Diabetol Metab Syndr. 2017 Apr 24;9:26.
21. Marques AP, et al. Dipeptidyl peptidase IV (DPP-IV) inhibition prevents fibrosis in adipose tissue of obese mice. Biochim Biophys Acta Gen Subj. 2018 Mar;1862(3):403-413.
22. Zilleßen P, et al. Metabolic role of dipeptidyl peptidase 4 (DPP4) in primary human (pre)adipocytes. Sci Rep. 2016 Mar 17;6:23074.
23. Han J, et al. ER stress signalling through eIF2α and CHOP, but not IRE1α, attenuates adipogenesis in mice. Diabetologia. 2013 Apr;56(4):911-24.
24. Kobori M, et al. Dietary Intake of Curcumin Improves eIF2 Signaling and Reduces Lipid Levels in the White Adipose Tissue of Obese Mice. Sci Rep. 2018 Jun 13;8(1):9081.
25. Dagon Y, Avraham Y, Berry EM. AMPK activation regulates apoptosis, adipogenesis, and lipolysis by eIF2alpha in adipocytes. Biochem Biophys Res Commun. 2006 Feb 3;340(1):43-7.
26. Inoue M, et al. Compartmentalization of the exocyst complex in lipid rafts controls Glut4 vesicle tethering. Mol Biol Cell. 2006 May;17(5):2303-11.
27. Lizunov VA, et al. Insulin regulates fusion of GLUT4 vesicles independent of Exo70-mediated tethering. J Biol Chem. 2009 Mar 20;284(12):7914-9.
28. Wang S, et al Inducible *Exoc7/Exo70* knockout reveals a critical role of the exocyst in insulin-regulated GLUT4 exocytosis. J Biol Chem. 2019 Dec 27;294(52):19988-19996.
29. Miao Y, et al. Novel adipokine asprosin modulates browning and adipogenesis in white adipose tissue. J Endocrinol. 2021 May;249(2):83-93.
30. Muthu ML, Reinhardt DP. Fibrillin-1 and fibrillin-1-derived asprosin in adipose tissue function and metabolic disorders. J Cell Commun Signal. 2020 Jun;14(2):159-173.
31. Passarge E, Robinson PN, Graul-Neumann LM. Marfanoid-progeroid-lipodystrophy syndrome: a newly recognized fibrillinopathy. Eur J Hum Genet. 2016 Aug;24(9):1244-7.
32. Takenouchi T, et al. Severe congenital lipodystrophy and a progeroid appearance: Mutation in the penultimate exon of FBN1 causing a recognizable phenotype. Am J Med Genet A. 2013 Dec;161A(12):3057-62.
33. Zhao L, et al. Fibroblast growth factor 1 ameliorates adipose tissue inflammation and systemic insulin resistance via enhancing adipocyte mTORC2/Rictor signal. J Cell Mol Med. 2020 Nov;24(21):12813-12825.
34. Sun K, Scherer PE. The PPARγ-FGF1 axis: an unexpected mediator of adipose tissue homeostasis. Cell Res. 2012 Oct;22(10):1416-8.
35. Wang S, et al. Adipocyte Piezo1 mediates obesogenic adipogenesis through the FGF1/FGFR1 signaling pathway in mice. Nat Commun. 2020 May 8;11(1):2303.
36. Jonker JW,et al. A PPARγ-FGF1 axis is required for adaptive adipose remodelling and metabolic homeostasis. Nature. 2012 May 17;485(7398):391-4.
37. Nies VJ, et al. Fibroblast Growth Factor Signaling in Metabolic Regulation. Front Endocrinol (Lausanne). 2016 Jan 19;6:193.
38. Lindegaard B, et al. Expression of fibroblast growth factor-21 in muscle is associated with lipodystrophy, insulin resistance and lipid disturbances in patients with HIV. PLoS One. 2013;8(3):e55632.
39. Wu L, et al. *GNPDA2* Gene Affects Adipogenesis and Alters the Transcriptome Profile of Human Adipose-Derived Mesenchymal Stem Cells. Int J Endocrinol. 2019 Aug 1;2019:9145452.
40. Chan CY, et al. Transcription factor HMG box-containing protein 1 (HBP1) modulates mitotic clonal expansion (MCE) during adipocyte differentiation. J Cell Physiol. 2018 May;233(5):4205-4215.
41. Zhang X, et al. HSPA12A is required for adipocyte differentiation and diet-induced obesity through a positive feedback regulation with PPARγ. Cell Death Differ. 2019 Nov;26(11):2253-2267.
42. Vietor I, et al. The negative adipogenesis regulator DLK1 is transcriptionally regulated by TIS7 (IFRD1) and translationally by its orthologue SKMc15 (IFRD2). BioRXiv Preprint, 2020 Oct 15.
43. Vietor I, et al. TIS7 and SKMc15 Regulate Adipocyte Differentiation and Intestinal Lipid Absorption. BioRXiv Preprint, 2019 Jul 30.
44. Moure R, et al. Levels of β-klotho determine the thermogenic responsiveness of adipose tissues: involvement of the autocrine action of FGF21. Am J Physiol Endocrinol Metab. 2021 Apr 1;320(4):E822-E834.
45. Ogawa Y, et al. BetaKlotho is required for metabolic activity of fibroblast growth factor 21. Proc Natl Acad Sci U S A. 2007 May 1;104(18):7432-7.
46. Simon MF, et al. Lysophosphatidic acid inhibits adipocyte differentiation via lysophosphatidic acid 1 receptor-dependent down-regulation of peroxisome proliferator-activated receptor gamma2. J Biol Chem. 2005 Apr 15;280(15):14656-62.
47. Chabowski DS, et al. Lysophosphatidic acid acts on LPA_1_ receptor to increase H_2_ O_2_ during flow-induced dilation in human adipose arterioles. Br J Pharmacol. 2018 Nov;175(22):4266-4280.
48. Wang J, et al. miR-30e reciprocally regulates the differentiation of adipocytes and osteoblasts by directly targeting low-density lipoprotein receptor-related protein 6. Cell Death Dis. 2013 Oct 10;4(10):e845.
49. Liu W, et al. Low density lipoprotein (LDL) receptor-related protein 6 (LRP6) regulates body fat and glucose homeostasis by modulating nutrient sensing pathways and mitochondrial energy expenditure. J Biol Chem. 2012 Mar 2;287(10):7213-23.
50. Zhao C, et al. MAT2B promotes adipogenesis by modulating SAMe levels and activating AKT/ERK pathway during porcine intramuscular preadipocyte differentiation. Exp Cell Res. 2016 May 15;344(1):11-21.
51. Li C, et al. Adipose-derived mesenchymal stem cells attenuate ischemic brain injuries in rats by modulating miR-21-3p/MAT2B signaling transduction. Croat Med J. 2019 Oct 31;60(5):439-448.
52. Kim JY, et al. ER Stress Drives Lipogenesis and Steatohepatitis via Caspase-2 Activation of S1P. Cell. 2018 Sep 20;175(1):133-145.e15.
53. Takahashi Y, et al. Perilipin-mediated lipid droplet formation in adipocytes promotes sterol regulatory element-binding protein-1 processing and triacylglyceride accumulation. PLoS One. 2013 May 29;8(5):e64605.
54. Ostrakhovitch EA, et al. 3-Mercaptopyruvate sulfurtransferase disruption in dermal fibroblasts facilitates adipogenic trans-differentiation. Exp Cell Res. 2019 Dec 15;385(2):111683.
55. Ying W, et al. MiR-690, an exosomal-derived miRNA from M2-polarized macrophages, improves insulin sensitivity in obese mice. Cell Metab. 2021 Apr 6;33(4):781-790.e5.
56. Navas LE, Carnero A. NAD^+^ metabolism, stemness, the immune response, and cancer. Signal Transduct Target Ther. 2021 Jan 1;6(1):2.
57. Katwan OJ, et al. AMP-activated protein kinase complexes containing the β2 regulatory subunit are up-regulated during and contribute to adipogenesis. Biochem J. 2019 Jun 26;476(12):1725-1740.
58. Ding Q, Wang Z, Chen Y. Endocytosis of adiponectin receptor 1 through a clathrin- and Rab5-dependent pathway. Cell Res. 2009 Mar;19(3):317-27.
59. Tessneer KL, et al. Rab5 activity regulates GLUT4 sorting into insulin-responsive and non-insulin-responsive endosomal compartments: a potential mechanism for development of insulin resistance. Endocrinology. 2014 Sep;155(9):3315-28.
60. Karvela A, et al. Adiponectin Signaling and Impaired GTPase Rab5 Expression in Adipocytes of Adolescents with Obesity. Horm Res Paediatr. 2020;93(5):287-296.
61. Xie L, O'Reilly CP, Chapes SK, Mora S. Adiponectin and leptin are secreted through distinct trafficking pathways in adipocytes. Biochim Biophys Acta. 2008 Feb;1782(2):99-108.
62. Chun KH, et al. Regulation of glucose transport by ROCK1 differs from that of ROCK2 and is controlled by actin polymerization. Endocrinology. 2012 Apr;153(4):1649-62.
63. Lee DH, et al. Targeted disruption of ROCK1 causes insulin resistance in vivo. J Biol Chem. 2009 May 1;284(18):11776-80.
64. Dankel SN, et al. The Rho GTPase RND3 regulates adipocyte lipolysis. Metabolism. 2019 Dec;101:153999.
65. Imai T, Jiang M, Chambon P, Metzger D. Impaired adipogenesis and lipolysis in the mouse upon selective ablation of the retinoid X receptor alpha mediated by a tamoxifen-inducible chimeric Cre recombinase (Cre-ERT2) in adipocytes. Proc Natl Acad Sci U S A. 2001 Jan 2;98(1):224-8.
66. Shoucri BM, et al. Retinoid X Receptor Activation During Adipogenesis of Female Mesenchymal Stem Cells Programs a Dysfunctional Adipocyte. Endocrinology. 2018 Aug 1;159(8):2863-2883.
67. Lefebvre B, et al. Proteasomal degradation of retinoid X receptor alpha reprograms transcriptional activity of PPARgamma in obese mice and humans. J Clin Invest. 2010 May;120(5):1454-68.
68. Garg A, et al. A gene for congenital generalized lipodystrophy maps to human chromosome 9q34. J Clin Endocrinol Metab. 1999 Sep;84(9):3390-4.
69. Mizuarai S, et al. Identification of dicarboxylate carrier Slc25a10 as malate transporter in de novo fatty acid synthesis. J Biol Chem. 2005 Sep 16;280(37):32434-41.
70. Fukunaka A, et al. Zinc transporter ZIP13 suppresses beige adipocyte biogenesis and energy expenditure by regulating C/EBP-β expression. PLoS Genet. 2017 Aug 30;13(8):e1006950.
71. Liu C, et al. Fat-Specific Knockout of Mecp2 Upregulates Slpi to Reduce Obesity by Enhancing Browning. Diabetes. 2020 Jan;69(1):35-47.
72. Adapala VJ, Buhman KK, Ajuwon KM. Novel anti-inflammatory role of SLPI in adipose tissue and its regulation by high fat diet. J Inflamm (Lond). 2011 Feb 28;8:5.
73. Kim JH, J. C-terminus of HSC70-Interacting Protein (CHIP) Inhibits Adipocyte Differentiation via Ubiquitin- and Proteasome-Mediated Degradation of PPARγ. Sci Rep. 2017 Jan 6;7:40023.
74. Lim CY, et al. Tropomodulin3 is a novel Akt2 effector regulating insulin-stimulated GLUT4 exocytosis through cortical actin remodeling. Nat Commun. 2015 Jan 9;6:5951.
75. Zhang Y, Gu M, Ma Y, Peng Y. LncRNA TUG1 reduces inflammation and enhances insulin sensitivity in white adipose tissue by regulating miR-204/SIRT1 axis in obesity mice. Mol Cell Biochem. 2020 Dec;475(1-2):171-183.
76. Long J, et al. Role for carbohydrate response element-binding protein (ChREBP) in high glucose-mediated repression of long noncoding RNA Tug1. J Biol Chem. 2020 Nov 20;295(47):15840-15852.
77. Zhang Y, Ma Y, Gu M, Peng Y. lncRNA TUG1 promotes the brown remodeling of white adipose tissue by regulating miR‑204‑targeted SIRT1 in diabetic mice. Int J Mol Med. 2020 Dec;46(6):2225-2234.
78. Peterson JM, et al. CTRP2 overexpression improves insulin and lipid tolerance in diet-induced obese mice. PLoS One. 2014 Feb 20;9(2):e88535.
79. Lei X, Wong GW. C1q/TNF-related protein 2 (CTRP2) deletion promotes adipose tissue lipolysis and hepatic triglyceride secretion. J Biol Chem. 2019 Oct 25;294(43):15638-15649.
80. Ou CY, et al. Coregulator cell cycle and apoptosis regulator 1 (CCAR1) positively regulates adipocyte differentiation through the glucocorticoid signaling pathway. J Biol Chem. 2014 Jun 13;289(24):17078-86.
81. Moreno-Navarrete JM, et al. Deleted in breast cancer 1 plays a functional role in adipocyte differentiation. Am J Physiol Endocrinol Metab. 2015 Apr 1;308(7):E554-61.
82. Escande C, et al. Deleted in breast cancer 1 limits adipose tissue fat accumulation and plays a key role in the development of metabolic syndrome phenotype. Diabetes. 2015 Jan;64(1):12-22.
83. Moreno-Navarrete JM, et al. DBC1 is involved in adipocyte inflammation and is a possible marker of human adipose tissue senescence. Obesity (Silver Spring). 2015 Mar;23(3):519-22.
84. Able AA, Richard AJ, Stephens JM. Loss of DBC1 (CCAR2) affects TNFα-induced lipolysis and *Glut4* gene expression in murine adipocytes. J Mol Endocrinol. 2018 Oct 15;61(4):195-205.
85. Botero V, et al. Neurofibromin regulates metabolic rate via neuronal mechanisms in Drosophila. Nat Commun. 2021 Jul 13;12(1):4285.
86. Souza MLR, et al. Increased resting metabolism in neurofibromatosis type 1. Clin Nutr ESPEN. 2019 Aug;32:44-49.
87. Summers MA, et al. Dietary intervention rescues myopathy associated with neurofibromatosis type 1. Hum Mol Genet. 2018 Feb 15;27(4):577-588.
88. Wei X, et al. Cell autonomous requirement of neurofibromin (Nf1) for postnatal muscle hypertrophic growth and metabolic homeostasis. J Cachexia Sarcopenia Muscle. 2020 Dec;11(6):1758-1778.
89. Greenberg CC, Danos AM, Brady MJ. Central role for protein targeting to glycogen in the maintenance of cellular glycogen stores in 3T3-L1 adipocytes. Mol Cell Biol. 2006 Jan;26(1):334-42.
90. Printen JA, Brady MJ, Saltiel AR. PTG, a protein phosphatase 1-binding protein with a role in glycogen metabolism. Science. 1997 Mar 7;275(5305):1475-8.
91. Matsui Y, et al. Overexpression of TNF-α converting enzyme promotes adipose tissue inflammation and fibrosis induced by high fat diet. Exp Mol Pathol. 2014 Dec;97(3):354-8.
92. Yong SB, Song Y, Kim YH. Visceral adipose tissue macrophage-targeted TACE silencing to treat obesity-induced type 2 diabetes. Biomaterials. 2017 Dec;148:81-89.
93. Menghini R, et al. The role of ADAM17 in metabolic inflammation. Atherosclerosis. 2013 May;228(1):12-7.
94. Wang Y, Sul HS. Ectodomain shedding of preadipocyte factor 1 (Pref-1) by tumor necrosis factor alpha converting enzyme (TACE) and inhibition of adipocyte differentiation. Mol Cell Biol. 2006 Jul;26(14):5421-35.
95. Gelling RW, et al. Deficiency of TNFalpha converting enzyme (TACE/ADAM17) causes a lean, hypermetabolic phenotype in mice. Endocrinology. 2008 Dec;149(12):6053-64.
96. Lownik JC, et al. Adipocyte ADAM17 plays a limited role in metabolic inflammation. Adipocyte. 2020 Dec;9(1):509-522.
97. Takahashi A, et al. The CCR4-NOT Deadenylase Complex Maintains Adipocyte Identity. Int J Mol Sci. 2019 Oct 24;20(21):5274.
98. Deconinck AE, et al. Utrophin-dystrophin-deficient mice as a model for Duchenne muscular dystrophy. Cell. 1997 Aug 22;90(4):717-27.
