# Supplementary material for: Systems genetics analysis of human body fat distribution genes identifies adipocyte processes
Source: Life Sci Alliance. 2024 May 3;7(7):e202402603. doi: 10.26508/lsa.202402603 (PMC11068934; doi:10.26508/lsa.202402603)
Supplement: Supplementary file 9 [file LSA-2024-02603_Supplemental_Data_2.docx]

**Extended Bibliography 2: Evidence of Key Driver Involvement in Wnt Signaling**

1. Ganner A, et al. Regulation of ciliary polarity by the APC/C. Proc Natl Acad Sci U S A. 2009 Oct 20;106(42):17799-804.
2. Chen D, et al. ANTXR1, a stem cell-enriched functional biomarker, connects collagen signaling to cancer stem-like cells and metastasis in breast cancer. Cancer Res. 2013 Sep 15;73(18):5821-33.
3. Cheng B, et al. The role of anthrax toxin protein receptor 1 as a new mechanosensor molecule and its mechanotransduction in BMSCs under hydrostatic pressure. Sci Rep. 2019 Sep 2;9(1):12642.
4. Verma K, Gu J, Werner E. Tumor endothelial marker 8 amplifies canonical Wnt signaling in blood vessels. PLoS One. 2011;6(8):e22334.
5. Ding C, et al. Tumor Endothelial Marker 8 Promotes Proliferation and Metastasis *via* the Wnt/β-Catenin Signaling Pathway in Lung Adenocarcinoma. Front Oncol. 2021 Oct 14;11:712371.
6. Sheen VL, et al. Mutations in ARFGEF2 implicate vesicle trafficking in neural progenitor proliferation and migration in the human cerebral cortex. Nat Genet. 2004 Jan;36(1):69-76.
7. Li CC, et al. Enhancement of β-catenin activity by BIG1 plus BIG2 via Arf activation and cAMP signals. Proc Natl Acad Sci U S A. 2016 May 24;113(21):5946-51.
8. Lalli MA, et al. Haploinsufficiency of BAZ1B contributes to Williams syndrome through transcriptional dysregulation of neurodevelopmental pathways. Hum Mol Genet. 2016 Apr 1;25(7):1294-306.
9. Schepeler T, et al. Attenuation of the beta-catenin/TCF4 complex in colorectal cancer cells induces several growth-suppressive microRNAs that target cancer promoting genes. Oncogene. 2012 May 31;31(22):2750-60.
10. Jiang S, et al. KIAA1522 Promotes the Progression of Hepatocellular Carcinoma via the Activation of the Wnt/β-Catenin Signaling Pathway. Onco Targets Ther. 2020 Jun 16;13:5657-5668.
11. Silhankova M, et al. Wnt signalling requires MTM-6 and MTM-9 myotubularin lipid-phosphatase function in Wnt-producing cells. EMBO J. 2010 Dec 15;29(24):4094-105.
12. Chen H, et al. REGγ accelerates melanoma formation by regulating Wnt/β-catenin signalling pathway. Exp Dermatol. 2017 Nov;26(11):1118-1124.
13. Li L, et al. REGγ is critical for skin carcinogenesis by modulating the Wnt/β-catenin pathway. Nat Commun. 2015 Apr 24;6:6875.
14. Essien BE, et al. Transcription Factor ZBP-89 Drives a Feedforward Loop of β-Catenin Expression in Colorectal Cancer. Cancer Res. 2016 Dec 1;76(23):6877-6887.
15. Ocadiz-Ruiz R, et al. ZBP-89 function in colonic stem cells and during butyrate-induced senescence. Oncotarget. 2017 Oct 9;8(55):94330-94344.
16. Mirra S, et al. Function of Armcx3 and Armc10/SVH Genes in the Regulation of Progenitor Proliferation and Neural Differentiation in the Chicken Spinal Cord. Front Cell Neurosci. 2016 Mar 3;10:47.
17. Pan M, et al. BNIP-2 retards breast cancer cell migration by coupling microtubule-mediated GEF-H1 and RhoA activation. Sci Adv. 2020 Jul 31;6(31):eaaz1534.
18. Soh UJ, Low BC. BNIP2 extra long inhibits RhoA and cellular transformation by Lbc RhoGEF via its BCH domain. J Cell Sci. 2008 May 15;121(Pt 10):1739-49.
19. Wen J, et al. IL-8 promotes cell migration through regulating EMT by activating the Wnt/β-catenin pathway in ovarian cancer. J Cell Mol Med. 2020 Jan;24(2):1588-1598.
20. Ruffner H, et al. R-Spondin potentiates Wnt/β-catenin signaling through orphan receptors LGR4 and LGR5. PLoS One. 2012;7(7):e40976.
21. Lee S, et al. WNT Signaling Driven by R-spondin 1 and LGR6 in High-grade Serous Ovarian Cancer. Anticancer Res. 2020 Nov;40(11):6017-6028.
22. Gore AV, et al. Rspo1/Wnt signaling promotes angiogenesis via Vegfc/Vegfr3. Development. 2011 Nov;138(22):4875-86.
23. Geng A, et al. A novel function of R-spondin1 in regulating estrogen receptor expression independent of Wnt/β-catenin signaling. Elife. 2020 Aug 4;9:e56434.
24. Binnerts ME, et al. R-Spondin1 regulates Wnt signaling by inhibiting internalization of LRP6. Proc Natl Acad Sci U S A. 2007 Sep 11;104(37):14700-5.
25. Chen D, et al. TYRO3 facilitates cell growth and metastasis via activation of the Wnt/β-catenin signaling pathway in human gastric cancer cells. Aging (Albany NY). 2020 Feb 4;12(3):2261-2274.
26. Al Kafri N, Hafizi S. Galectin-3 Stimulates Tyro3 Receptor Tyrosine Kinase and Erk Signalling, Cell Survival and Migration in Human Cancer Cells. Biomolecules. 2020 Jul 11;10(7):1035.
27. Zhu YZ, et al. Inhibition of TYRO3/Akt signaling participates in hypoxic injury in hippocampal neurons. Neural Regen Res. 2016 May;11(5):752-7.
