# Supplementary material for: Systems genetics analysis of human body fat distribution genes identifies adipocyte processes
Source: Life Sci Alliance. 2024 May 3;7(7):e202402603. doi: 10.26508/lsa.202402603 (PMC11068934; doi:10.26508/lsa.202402603)
Supplement: Supplementary file 10 [file LSA-2024-02603_Supplemental_Data_3.docx]

**Extended Bibliography 3: Evidence of Key Driver Involvement in Mitochondrial Activity**

1. Feng H, et al. CTRP3 promotes energy production by inducing mitochondrial ROS and up-expression of PGC-1α in vascular smooth muscle cells. Exp Cell Res. 2016 Feb 15;341(2):177-86.
2. Gao J, Qian T, Wang W. CTRP3 Activates the AMPK/SIRT1-PGC-1α Pathway to Protect Mitochondrial Biogenesis and Functions in Cerebral Ischemic Stroke. Neurochem Res. 2020 Dec;45(12):3045-3058.
3. Zhang CL, et al. Globular CTRP3 promotes mitochondrial biogenesis in cardiomyocytes through AMPK/PGC-1α pathway. Biochim Biophys Acta Gen Subj. 2017 Jan;1861(1 Pt A):3085-3094.
4. Xie Y, et al. The proteasome activator REGγ accelerates cardiac hypertrophy by declining PP2Acα-SOD2 pathway. Cell Death Differ. 2020 Oct;27(10):2952-2972.
5. Moncsek A, et al. Evidence for anti-apoptotic roles of proteasome activator 28γ via inhibiting caspase activity. Apoptosis. 2015 Sep;20(9):1211-28.
6. Gustafsson ÅB, Dorn GW 2nd. Evolving and Expanding the Roles of Mitophagy as a Homeostatic and Pathogenic Process. Physiol Rev. 2019 Jan 1;99(1):853-892.
7. Metzger MB, et al. A protein quality control pathway at the mitochondrial outer membrane. Elife. 2020 Mar 2;9:e51065.
8. Emfinger CH, et al. β Cell-specific deletion of Zfp148 improves nutrient-stimulated β cell Ca2+ responses. JCI Insight. 2022 May 23;7(10):e154198.
9. Hartmann B, et al. Homozygous YME1L1 mutation causes mitochondriopathy with optic atrophy and mitochondrial network fragmentation. Elife. 2016 Aug 6;5:e16078.
10. Ruan Y, et al. Loss of Yme1L perturbates mitochondrial dynamics. Cell Death Dis. 2013 Oct 31;4(10):e896.
11. MacVicar T, et al. Lipid signalling drives proteolytic rewiring of mitochondria by YME1L. Nature. 2019 Nov;575(7782):361-365.
12. Gowans GJ, et al. INO80 Chromatin Remodeling Coordinates Metabolic Homeostasis with Cell Division. Cell Rep. 2018 Jan 16;22(3):611-623.
13. Diquigiovanni C, et al. A novel mutation in *SPART* gene causes a severe neurodevelopmental delay due to mitochondrial dysfunction with complex I impairments and altered pyruvate metabolism. FASEB J. 2019 Oct;33(10):11284-11302.
14. Seo BA, et al. TRIP12 ubiquitination of glucocerebrosidase contributes to neurodegeneration in Parkinson's disease. Neuron. 2021 Dec 1;109(23):3758-3774.e11.
15. Maruyama H, et al. Medullary thick ascending limb impairment in the Gla^tm^Tg(CAG-A4GALT) Fabry model mice. FASEB J. 2018 Aug;32(8):4544-4559.
16. Serrat R, et al. The non-canonical Wnt/PKC pathway regulates mitochondrial dynamics through degradation of the arm-like domain-containing protein Alex3. PLoS One. 2013 Jul 2;8(7):e67773.
17. Mou Z, Tapper AR, Gardner PD. The armadillo repeat-containing protein, ARMCX3, physically and functionally interacts with the developmental regulatory factor Sox10. J Biol Chem. 2009 May 15;284(20):13629-13640.
18. Howells CC, et al. The Bcl-2-associated death promoter (BAD) lowers the threshold at which the Bcl-2-interacting domain death agonist (BID) triggers mitochondria disintegration. J Theor Biol. 2011 Feb 21;271(1):114-23.
19. Liu XM, et al. Mitochondrial Function Regulated by Mitoguardin-1/2 Is Crucial for Ovarian Endocrine Functions and Ovulation. Endocrinology. 2017 Nov 1;158(11):3988-3999.
20. Liu XM, et al. Mitoguardin-1 and -2 promote maturation and the developmental potential of mouse oocytes by maintaining mitochondrial dynamics and functions. Oncotarget. 2016 Jan 12;7(2):1155-67.
21. Xu L, et al. Miga-mediated endoplasmic reticulum-mitochondria contact sites regulate neuronal homeostasis. Elife. 2020 Jul 10;9:e56584.
22. Deng L, et al. NMT1 inhibition modulates breast cancer progression through stress-triggered JNK pathway. Cell Death Dis. 2018 Nov 16;9(12):1143.
